# Discovery of C-12 dithiocarbamate andrographolide analogue as a novel antioxidant and α-glucosidase inhibitors: *In vitro* and *in silico* studies

**DOI:** 10.1371/journal.pone.0334026

**Published:** 2025-10-22

**Authors:** Utid Suriya, Chanathip Duangtha, Thanchanok Dontricharoen, Paveena Yamanont, Sakonwan Kuhaudomlarp, Prawit Thitiyanuwat, Bodee Nutho, Patcharee Arsakhant, Rungnapha Saeeng, Noppawan Phumala Morales, Supachoke Mangmool, Sutharinee Likitnukul

**Affiliations:** 1 Department of Biochemistry, Faculty of Science, Mahidol University, Bangkok, Thailand; 2 Department of Pharmacology, Faculty of Science, Mahidol University, Bangkok, Thailand; 3 Department of Chemistry and Center for Innovation in Chemistry, Faculty of Science, Burapha University, Chonburi, Thailand; 4 Department of Pharmaceutical Care, Faculty of Pharmacy, Chiang Mai University, Chiang Mai, Thailand; University of Chicago, UNITED STATES OF AMERICA

## Abstract

Type 2 diabetes mellitus (T2DM) is a global health issue associated with oxidative stress, inflammation, and insulin resistance. Even though α-glucosidase inhibitors such as acarbose are used in treatment, their efficacy is limited by gastrointestinal side effects. In this study, we evaluated the antioxidant properties and α-glucosidase inhibition of C-12 dithiocarbamate andrographolide analogues compared to the parent compound, andrographolide. Among all analogues, compound **3f** exhibited strong antioxidant activity, achieving 84% DPPH inhibition and a reducing antioxidant power activity of 254 μM ascorbic acid equivalent (AAE) at 500 μM. Additionally, molecular docking suggested a favorable binding to both yeast and human α-glucosidase at a comparable level as andrographolide, verified by the surface plasmon resonance (SPR) detection system, indicating a strong binding affinity with a dissociation constant (*K*_D_) of 12.86 μM. It also retains favorable physicochemical properties that align with drug-likeness based on Lipinski’s Rule. Functional assay confirmed its inhibitory activity with an IC_50_ of 411 μM against the yeast α-glucosidase enzyme model, which was greater than both andrographolide and acarbose. Further molecular dynamics (MD) simulation analysis revealed that compound **3f** exhibited stable and thermodynamically favorable binding to human α-glucosidase as well as interacting with key amino acids similar to those of andrographolide, providing a preliminary understanding of its potential relevance in a human enzyme context. Altogether, these findings highlight the significant potential of compound **3f** as a novel α-glucosidase inhibitor, offering a potential therapeutic alternative and paving the way for further anti-diabetic drug development.

## Introduction

Type 2 diabetes mellitus (T2DM) is a non-communicable disease (NCD) caused by insulin resistance, which leads to increased blood glucose levels. Pathophysiology of T2DM is associated with oxidative stress, chronic low-grade inflammation, and the effects of insulin resistance on normal glycemic control. Insulin resistance refers to an impairment of the response to the physiological function of insulin, which is crucial for stimulating glucose uptake in insulin target tissues, including skeletal muscle and adipose tissue. As a result, the pancreatic beta cells increase insulin production to compensate, eventually leading to a state of hyperinsulinemia [1]. Additionally, adipokines and pro-inflammatory cytokines secreted from excess adipose tissue contribute to the pathogenesis of T2DM by causing cellular oxidative stress [2]. Cellular damage occurs when the equilibrium between free radicals like reactive oxygen species (ROS) and antioxidants is imbalanced. Excessive ROS can damage proteins and lipids in cells, impairing insulin signaling and glucose metabolism. ROS also activates stress signaling pathways that degrade insulin receptor substrate (IRS) and interfere with the translocation of glucose transporter-4 (GLUT-4), directing it to lysosomes rather than the cell membrane, resulting in worsening hyperglycemia [3]. In T2DM, increased ROS generation combined with a diminished antioxidant capacity leads to oxidative damage and further decreases insulin sensitivity [4]. Moreover, elevated blood glucose levels lead to increased ROS production, resulting in lipid peroxidation, DNA damage, protein aggregation, and denaturation. This cascade ultimately stimulates apoptosis in pancreatic beta cells [5]. Patients with T2DM commonly exhibit key clinical signs such as excessive thirst (polydipsia), increased appetite (polyphagia), and frequent urination (polyuria) despite experiencing weight loss [6].

The primary goal of T2DM treatment is to control blood glucose levels and minimize oxidative stress in patients. Achieving glycemic control during both basal (fasting) and postprandial stages is crucial for preventing diabetic complications. α-glucosidase, a hydrolase enzyme that breaks down the α-1,4 glycosidic bonds, plays an important role in the carbohydrate absorption steps in the small intestine. This enzymatic activity impacts the postprandial blood glucose levels. Inhibition of α-glucosidase potentially attenuates the digestion of complex carbohydrates, resulting in reduced glucose absorption, which leads to blunted blood glucose levels after meals [7]. Currently, various oral hypoglycemic agents, which involve the target α-glucosidase enzyme and other mechanisms, are effectively used for pharmacological therapy in T2DM patients. However, some of these medications may have adverse effects and interact with other drugs. Medicinal plants have been utilized for therapeutic purposes for decades. Their extracts contain numerous bioactive compounds with antioxidant and anti-inflammatory activities that can be used to treat several diseases, including T2DM and its complications. *Andrographis paniculata* (Burm. F.) Nees is an herbal plant commonly used in Southeast Asia, particularly in Thai traditional medicine. Its primary bioactive compound is Andrographolide, a colorless crystalline bicyclic diterpenoid lactone [8]. Andrographolide is known for its numerous beneficial effects and may serve as an alternative herbal medicine due to its wide range of biological activities.

Andrographolide demonstrates the potential properties, including hepatoprotective effects, immunological benefits, antioxidant, and anti-inflammatory activities, and treatment for respiratory diseases, gastroenteritis, antidiarrheal, and intestinal issues [8,9]. Its pharmacological potential in addressing several health problems is primarily attributed to its ability to reduce ROS and inflammation [10–12]. Furthermore, the extract from *Andrographis paniculata* is also listed in the National List of Essential Medicine (NLEM) of Thailand for treating diarrhea and sore throat associated with the common cold. Additionally, numerous studies have reported the advantages of Andrographolide in improving the signs and symptoms of metabolic syndrome (MetS), such as lower blood glucose, cholesterol, and triglyceride levels [13–16]. The reduction in blood glucose levels results from the inhibition of glucose absorption in the intestine [17] and the upregulation of GLUT-4 in insulin-targeted tissues [13], though the precise mechanism of action requires further investigation. Furthermore, the positive impact of Andrographolide on lipid profiles, such as lowering triglyceride, total cholesterol, and low-density lipoprotein cholesterol (LDL-C), is directly associated with its ability to mitigate oxidative stress [18]. To further enhance its pharmacological potency, novel andrographolide analogues have been developed through modifications by acetylation of hydroxyl groups and the introduction of dithiocarbamate moieties at the C-12 position. These modifications enhance the biological activity of andrographolide analogues.

Herein, the derivatives of andrographolide accomplished by structural modification, adding dithiocarbamate at the C-12 position of andrographolide, were synthesized. Briefly, this process involved three steps reactions conducted in a single pot under mild conditions by *in-situ* generation of dithiocarbamate and addition at C-12 of andrographolide followed by elimination reactions, resulting in a diverse array of 12-dithiocarbamate-14-deoxyandrographolide derivatives [19]. Structural modification, particularly through the introduction of nucleophilic groups such as amino or dithiocarbamate moieties at the C-12 position of andrographolide, is an effective strategy for derivatization reported in previous literature. For example, Kasemsuk *et al.* [20] synthesized 12-*N*-substituted-14-deoxyandrographolide derivatives by introducing aniline moieties at C-12. These 12-amino analogues exhibited stronger cytotoxicity against P-388 and ASK cancer cells than the parent compound, and in some cases, even surpassed the potency of ellipticine. Arsakhant *et al*. [19] synthesized 12-dithiocarbamate derivatives, most of which showed selective cytotoxicity toward MCF-7 cells. Some derivatives were also tested as SARS-CoV-2 M^pro^ inhibitors, showing promising inhibitory activity better than the known inhibitor rutin and the parent andrographolide [21]. Collectively, these studies highlight C-12 as a key site for modification to improve anticancer and antiviral potency, supporting the design of novel andrographolide derivatives. Interestingly, dithiocarbamates possess significant physicochemical properties that enhance redox potential and have various medicinal applications, including anticancer, antibacterial, anti-Alzheimer’s, anti-inflammatory, and anti-diabetic activities [22]. Therefore, in this study, we aimed to identify the dithiocarbamate derivatives, potentially exhibiting dual properties of antioxidant and α-glucosidase inhibition compared to andrographolide. The combination of *in vitro* and *in silico* methods was conducted to accelerate “hit identification”, along with employing surface plasmon resonance (SPR), a biophysical method to experimentally validate the level of binding to α-glucosidase. This work identifies promising compounds and elucidates the detailed interactions and susceptibility toward ligand binding at the atomic level. The hit compound identified here could serve as promising candidates for further developing a novel anti-diabetic drug for T2DM management.

## Materials and methods

### Isolation of andrographolide and synthesis of C-12 dithiocarbamate andrographolide analogues

**Fig 1:**
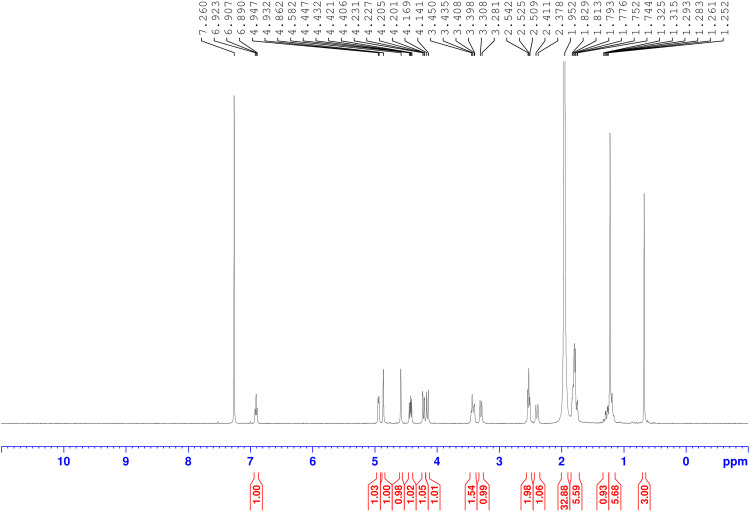
^1^H NMR spectrum of parent Andrographolide (initial compound for synthesis of dithiocarbamate-andrographolide analogues).

**Fig 2:**
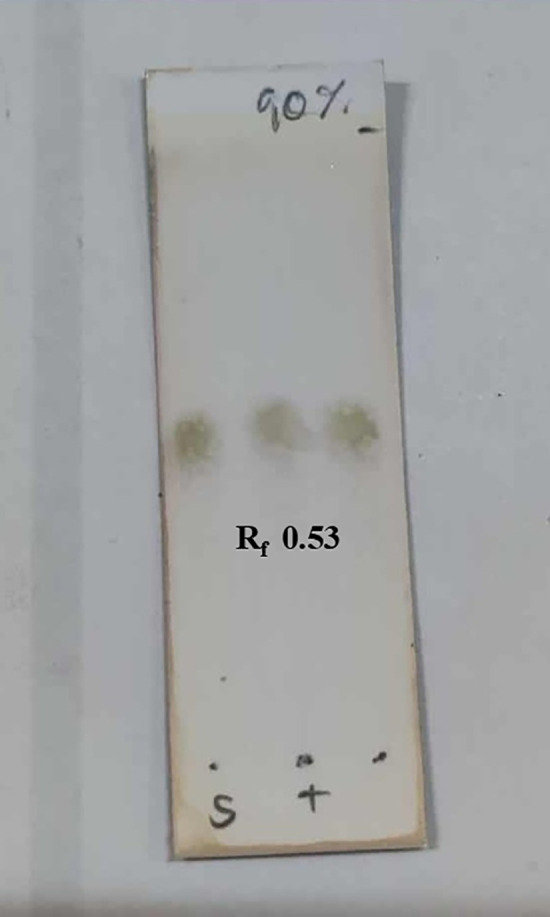
The TLC chromatogram of Andrographolide. Left: Andrographolide (standard); Right: Isolated Andrographolide from *Andrographis paniculata*; Middle: Co-spot of Left and Right compounds. TLC plate was observed under UV 254 nm. The mobile phase was 90% Ethyl acetate/*n*-hexane.

**Fig 3:**
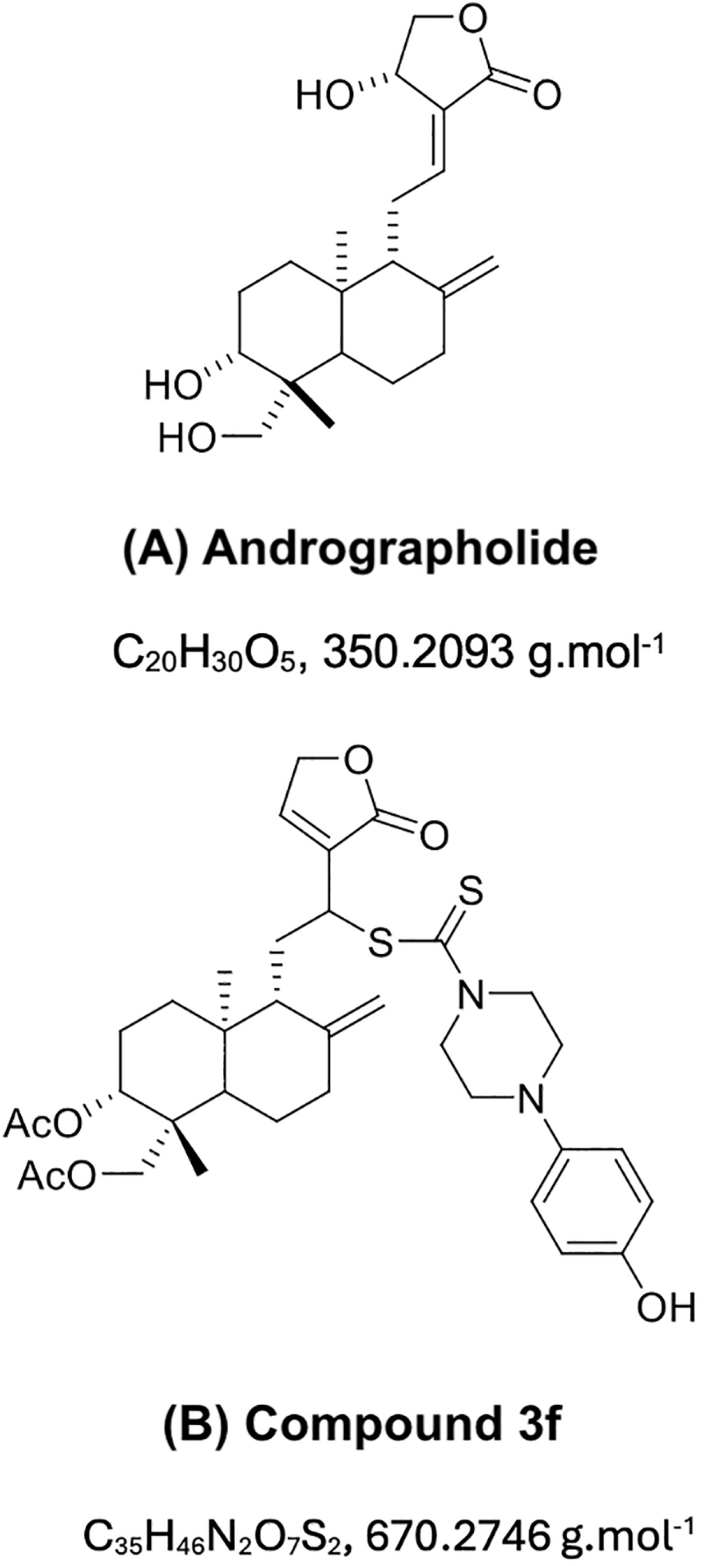
Chemical structure, formula, and molecular weight of the compounds. (A) Andrographolide, (B) compound **3f**.

Andrographolide was isolated from *Andrographis paniculata* with ethyl acetate at room temperature and then confirmed by both ^1^H NMR and thin-layer chromatography (TLC) compared with a standard andrographolide (Merck, Germany) (Figs 1–2). The extract was washed with hexane and dichloromethane and further recrystallized using methanol. The structural modifications for synthesizing andrographolide analogues were described in the previous study [19]. The chemical structures of andrographolide and the selected compound **3f** are shown below (Fig 3).

### Chemical reagents

All chemical reagents, including 1,1-Diphenyl-2-picrylhydrazyl (DPPH), α-tocopherol, ascorbic acid, 2,4,6-Tri(2-pyridyl)-s-triazine (TPTZ), ferrous sulfate (FeSO_4_), α-glucosidase (from *Saccharomyces cerevisiae*) (EC 3.2.1.20), *p*-nitrophenyl a-D-glucopyranoside (*p*-NPG) and acarbose were purchased from Sigma-Aldrich, St. Louis, MO, United States. CM5 chip was purchased from Cytiva. All other chemicals used in the experiment were of analytical grade, and ultrapure water was used throughout the chemical assays.

### DPPH radical scavenging activity

The DPPH radical scavenging activity was conducted to thoroughly evaluate the antioxidant potential of andrographolide and its analogues. We modified the assay employing the Sekhon-Loodu and Rupasinghe methods [23]. A DPPH solution was prepared in absolute ethanol, with a concentration of 200 μM. Next, 100 μL of DPPH solution was mixed with 100 μL of the sample in a 96-well plate. The mixture was then incubated for 30 minutes at room temperature. Following incubation, we measured the absorbance at 517 nm using a microplate reader. We tested the antioxidant properties of the compounds at a concentration of 500 μM to detect the weak-to-moderate antioxidants while addressing solubility limitations and minimizing assay artefacts, consistent with previous research [24]. The DPPH radical scavenging activity was precisely expressed as a percentage of DPPH inhibition (% DPPH Inhibition). We selected candidates based on their higher inhibitory activity and prepared various concentrations of these candidates for testing. α-tocopherol, also known as vitamin E, was employed as a positive control. The % DPPH Inhibition was calculated as follows:


$$\%\text{DPPH Inhibition}=\left(\text{1}–\left\{\frac{A}{B}\right\}\right)\times \text{100}$$

Where A was the absorbance of the DPPH with sample, and B was the absorbance of the DPPH with vehicle (blank).

### Ferric reducing antioxidant power (FRAP) activity

The FRAP activity assay was performed to assess the reducing power ability to reduce ferric ions (Fe^3+^) to ferrous ions (Fe^2+^), following the established method described by Sekhon-Loodu and Rupasinghe [23]. The FRAP reagent was freshly prepared by combining 300 mM acetate buffer pH 3.6, 10 mM 2,4,6- Tri(2-pyridyl)-s-triazine (TPTZ) dissolved in 40 mM HCl, and 20 mM FeCl_3_ in a ratio of 10:1:1. Ferrous sulfate (FeSO_4_) was used as a standard, with ascorbic acid serving as a control. The assay was conducted in a 96-well plate by mixing 20 μL of the sample, standard, or positive control with 80 μL of the FRAP solution. The mixture was then incubated for 20 minutes at room temperature, after which we measured absorbance at 595 nm using a microplate reader. The results were expressed as FRAP value, reported as ascorbic acid equivalent in μM (AAE, μM), providing a definitive measure of reducing power.

### Molecular docking

Molecular docking was performed to predict the ligand binding affinity with α-glucosidase in two different species, human and yeast. For yeast α-glucosidase, the three-dimensional structure was built from its amino acid sequence retrieved from Uniprot (ID: P38158, MAL32 gene) using AlphaFold 3 (S1 Fig) [25]. Then, the ligand binding site was computationally identified by the Fpocket (S2 Fig) [26]. For human α-glucosidase, there is an available X-ray crystal structure of the C-terminal subunit of human maltase-glucoamylase (MGAM) in complex with acarbose; thus, retrieved from the RCSB Protein Data Bank (PDB ID: 3TOP [27]). The binding site of the co-crystallized acarbose was used as the center for molecular docking, with coordinates set at x = −30.62, y = 35.65, and z = 26.44. A docking grid box of 20 Å × 20 Å × 20 Å was defined around this center. The 3D structures of andrographolide and compound **3f** were obtained from a previous study [21]. To validate the docking protocol, the co-crystallized inhibitor was first redocked into its original binding site, and the resulting conformation was compared to the experimental pose (S4 Fig). All protein and ligand structures were converted to PDBQT format using AutoDockTools version 1.5.7. Docking simulations were then carried out using AutoDock Vina XB [28] on a Linux operating system. The pose with the lowest binding energy (i.e., the best Vina XB score) was selected for further analysis.

### Drug-likeness and toxicity prediction

The focused physicochemical properties and drug-likeness based on Lipinski’s Rule were predicted using the SwissADME’s web-based tool (www.swissadme.ch/) [29–31]. The screened compound’s features were calculated compared to the parent compound, andrographolide.

### Surface plasmon resonance

All experiments were conducted using a Biacore X100 instrument at 25 °C with a running buffer of 1X PBS-P+ (10 mM phosphate buffer pH 7.4, 2.7 mM KCl, 137 mM NaCl, 0.05% Tween 20) supplemented with 5% DMSO. Compounds were initially dissolved in DMSO to a final concentration of 14 mM. The stock solution was then diluted to 700 μM in 1.05X PBS-P+ to prepare a compound solution containing 1X PBS-P+ with 5% DMSO. This compound solution was utilized to prepare the required concentrations for injections into the Biacore system. α-glucosidase from *Saccharomyces cerevisiae* was immobilized onto a CM5 chip following standard amine coupling procedures: the CM5 chip, containing channel 1 and 2, was activated with an injection of NHS/EDC mixture with a contact time of 540 s at a flow rate of 10 µL/min. This was followed by multiple injections of α-glucosidase dissolved in 10 mM sodium acetate pH 4.0 (50 µg/mL) onto channel 2, also with a contact time of 540 s at a flow rate of 10 µL/min. Both channels were inactivated by an injection of 1M ethanolamine at a flow rate of 10 µL/min for 540 s. An immobilization level of 3300 RU of α-glucosidase was achieved.

Multi-cycle affinity studies were conducted with a 100 s association phase and a 60 s dissociation phase, using a flow rate of 30 µL/min. Compounds were injected onto the chip at increasing concentrations of 7, 21, 70, 210, and 490 μM. Binding measurements were taken after reference subtraction of channel 1 (no immobilized α-glucosidase) and after subtracting the results from a blank injection with zero analyte concentration. All data evaluation was performed with Biacore X100 evaluation software (version 2.0).

### α-glucosidase inhibition activity

An *in vitro* α-glucosidase inhibitory assay is commonly used to evaluate the inhibitory effects of various compounds on the enzyme α-glucosidase, which is involved in carbohydrate digestion. Inhibiting this enzyme results in slower glucose absorption, which can be beneficial for managing postprandial blood sugar levels in diabetic conditions. This assay was modified from the methods described by Yusuf *et al*. [32]. The procedure involved mixing 25 μL of the sample, 150 μL of phosphate buffer saline (PBS) pH 7.4, and 75 μL of 10 mM *p*-NPG. The mixture was incubated at 37 °C for 10 min. Next, 50 μL of α-glucosidase enzyme from *Saccharomyces cerevisiae* at a concentration of 0.1 U/mL was added, then incubated at 37 °C for 10 min. To terminate the reaction, 500 μL of 0.1 M sodium carbonate (Na_2_CO_3_) was added to the mixture. The absorbance was measured using a microplate reader. The percentage of α-glucosidase inhibition was calculated as follows:


$$\alpha -\text{glucosidase inhibition}(\%)=\left(\text{1}–\left\{\frac{A-B}{C-D}\right\}\right)\times \text{100}$$

Where A was the absorbance of the sample with α-glucosidase enzyme, B was the absorbance of the sample, C was the absorbance of vehicle with α-glucosidase enzyme, and D was the absorbance of the vehicle (blank).

### Molecular dynamics (MD) simulations

The best-docked poses of andrographolide and compound **3f** in complex with MGAM were selected as the initial structures for molecular dynamics (MD) simulations using the AMBER22 software suite. The protonation states of ionizable amino acid residues were assigned at pH 7.4 using the PDB2PQR web server [33]. Ligand partial atomic charges were calculated with the Antechamber module in AMBER22, and other ligand parameters were derived from the General AMBER Force Field 2 (GAFF2). The protein was treated with the AMBER ff14SB force field [34]. The TIP3P water model [35] was used to solvate the system. MD simulations were performed under periodic boundary conditions in the isothermal-isobaric (*NPT*) ensemble at 310 K and 1 atm. Initial energy minimization was conducted in two stages: first, hydrogen atoms and water molecules were minimized using 500 steps of the steepest descent (SD) method, followed by 1,500 steps of the conjugate gradient (CG) method; then, the entire complex underwent the same minimization procedure. Electrostatic interactions were treated using the particle mesh Ewald (PME) method, and bonds involving hydrogen atoms were constrained with the SHAKE algorithm [36,37]. Temperature control was achieved using the Langevin thermostat, and pressure was maintained using the Berendsen barostat [38–40]. The production phase of the MD simulation was carried out for 300 ns with a 2-ns time step. Trajectory analysis was performed using the CPPTRAJ module, and per-residue energy decomposition analysis was conducted using the MM-GBSA method. The binding affinities of the ligands were estimated by calculating the end-point binding free energy (Δ*G*_bind_) using both MM-GBSA and MM-PBSA approaches [41].

### Statistical analysis

All the experiment results were performed in at least three independent experiments. The data are expressed as a mean ± standard deviation (SD). Significant values were considered as *p* ≤ 0.05. Analysis of statistically significant differences among groups was performed using one-way analysis of variance (ANOVA) with Bonferroni *post hoc* test. Data analysis was evaluated with GraphPad Prism 10.3.0 (GraphPad Software, San Diego, CA, United States).

## Results and discussion

### *In vitro* antioxidant screenings

Cellular damage occurs when the equilibrium between free radicals like reactive oxygen species (ROS) and antioxidants is imbalanced. Excessive ROS can damage proteins and lipids within cells, impairing insulin signaling and glucose metabolism, which are critical in the development of diabetes-related complications. Therefore, this study evaluated the antioxidant capacities and α-glucosidase inhibitory activities of andrographolide analogues to assess their potential as candidates for anti-diabetic agents.

### DPPH radical scavenging activity

**Fig 4:**
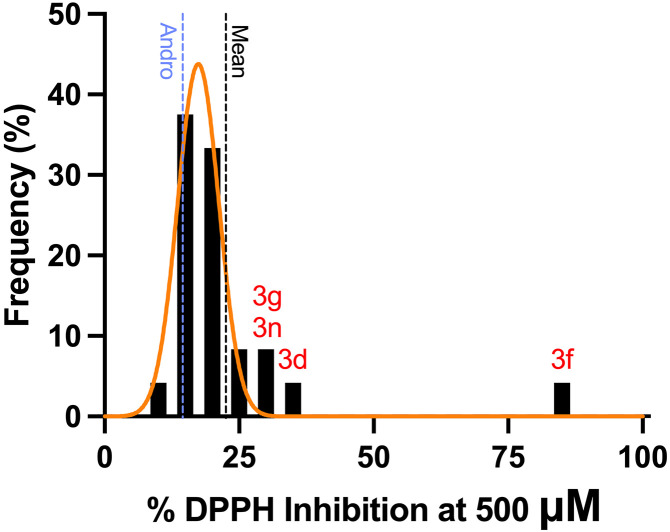
DPPH radical scavenging capacity of andrographolide and analogues.

To evaluate the antioxidative properties of the compounds on radical scavenging capacity using the DPPH assay. This assay effectively demonstrates the compounds’ ability to donate hydrogen atoms or electrons to neutralize free radicals, resulting in a significant decrease in absorbance. We tested a total of 23 compounds, including crude extract, andrographolide, and its analogues, for their DPPH radical scavenging activity. The results, indicated as %DPPH inhibition, ranged from 12.07% to an impressive 84.47%, with an average of 22.50%. Compound 3f exhibited the highest activity at the inhibition rate of 84.47 ± 1.14% (Fig 4), while the other compounds displayed results closer to the average. The antioxidant activity results of all tested compounds were presented in supplementary data: S1 Table.

**Fig 5:**
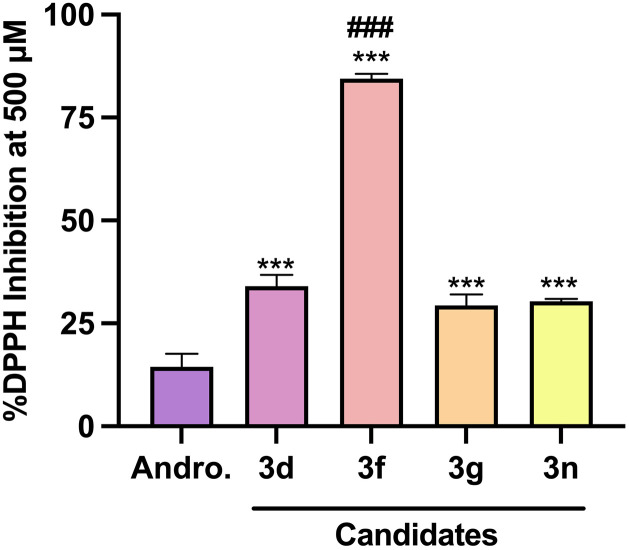
% DPPH inhibition at 500 μM of andrographolide and candidates. Data are presented as mean ± SD (n = 3). Statistical significance was performed by one-way ANOVA followed by Bonferroni *post hoc* multiple comparisons. *** represents statistical significance compared to andrographolide (Andro.) (*p* < 0.001). ^###^ represents statistical significance among all candidates. (*p* < 0.001).

**Fig 6:**
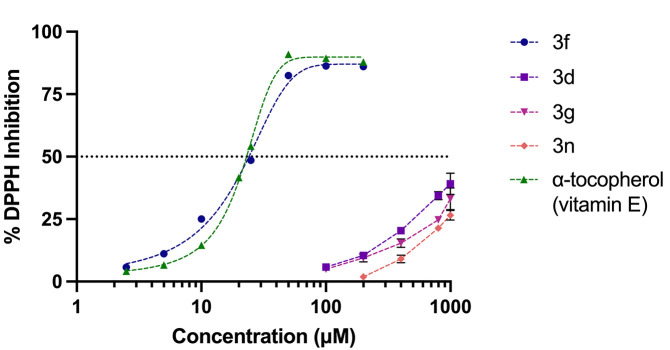
DPPH inhibition curves of α-tocopherol and compounds 3f, 3d, 3g, and 3n. Data are presented as mean ± SD (n = 3).

At a concentration of 500 μM, all candidates, including compounds 3d, 3f, 3g, and 3n, exhibited a percentage DPPH Inhibition that was significantly higher than that of the parent compound, andrographolide (Fig 5). We tested various concentrations of the candidates with a 200 μM DPPH solution, and compound 3f clearly demonstrated a significant scavenging capacity compared to other candidates. It achieved an IC_50_ value similar to that of α-tocopherol, at approximately 25 μM, whereas the other tested analogues could not reach 50% inhibition, even at the highest concentration tested (Fig 6).

### Ferric reducing antioxidant power (FRAP)

**Fig 7:**
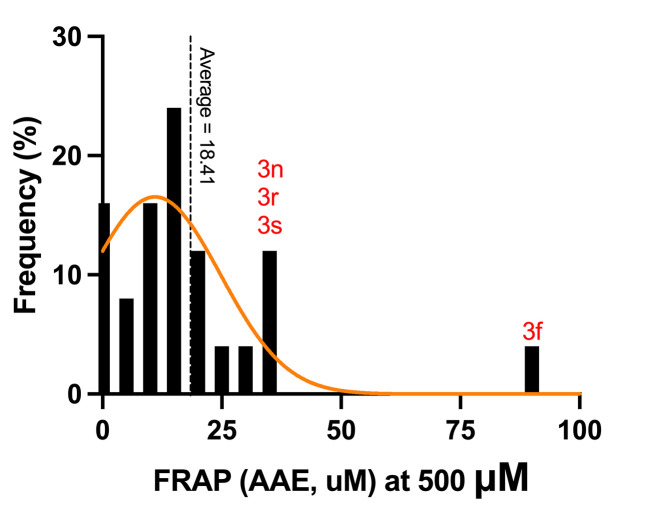
FRAP activity of andrographolide and the tested analogues.

**Fig 8:**
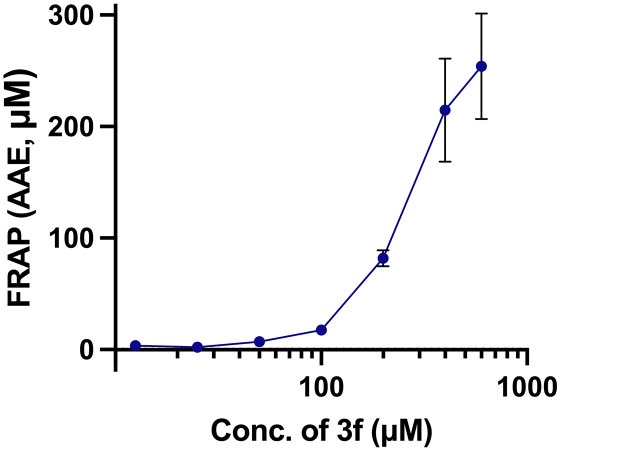
FRAP activity of compound 3f at a concentration range up to 600 μM. Data are presented as mean ± SD (n=3).

The FRAP assay evaluates the reducing power of antioxidants by measuring their ability to reduce ferric ions (Fe^3+^) to ferrous ions (Fe^2+^). In this study, we measured the FRAP activity as an ascorbic acid equivalent (AAE) in μM, highlighting its effectiveness in reducing ferric ions, a vital metal ion involved in a crucial role in oxidative processes. We found that FRAP activity was significant among andrographolide and its analogues. At a concentration of 500 μM, the average FRAP activity of andrographolide, and its analogues was 18.41 AAE. Notably, compound 3f exhibited the highest activity at 88.30 ± 21.45 AAE (Fig 7). The FRAP results of all tested compounds were presented in the supplementary data: S2 Table. Additionally, compound 3f demonstrated the highest FRAP activity, reaching 253.90 ± 47.40 AAE at a concentration of 600 μM (Fig 8), showcasing its antioxidant potential. This reinforces the potential of these compounds in the realm of oxidative stress reduction.

The antioxidant activity of andrographolide and the selected candidates, as shown by DPPH and FRAP assays, was particularly notable for compound **3f**, which exhibited greater antioxidant activity. Structurally, compound **3f** features a substituted phenolic moiety linked via a piperazine bridge to a dithiocarbamate group. This combination likely augments its electron-donating capacity compared to the parent andrographolide. Phenolic groups are recognized as effective radical scavengers due to their propensity to donate hydrogen atoms, thereby neutralizing free radicals. The resulting phenoxyl radical is resonance-stabilized over the aromatic ring, diminishing its reactivity and disrupting radical chain reactions. This enhanced stabilization may underlie the improved biological activity observed for compound **3f**. Consequently, the molecular structure of compound **3f** appears to promote electron donation, facilitate radical stabilization, and strengthen overall antioxidant activity. Reduction of oxidative stress was closely associated with their anti-diabetic properties. Oxidative stress plays a significant role in insulin resistance and beta-cell dysfunction [42]. By mitigating oxidative damage, andrographolide and the selected analogues may improve insulin sensitivity and preserve pancreatic beta-cell function, thereby contributing to improve glucose regulation.

### *In silico* α-glucosidase inhibition

#### Molecular docking.

To preliminarily investigate the inhibitory potential of compound **3f** against α-glucosidase, alongside its previously noted strong antioxidative properties, molecular docking was conducted to predict its binding affinity and intermolecular interactions, using andrographolide as a reference compound. Docking simulations were performed with α-glucosidase from both yeast and human strains. Due to the unavailability of purified human α-glucosidase, subsequent experimental validation in this study was conducted on the yeast enzyme model. Docking results, against yeast α-glucosidase, revealed that compound **3f** exhibited a binding affinity comparable to its parent compound, andrographolide, with calculated binding energies of –8.9 and –9.1 kcal/mol, respectively. To assess the translational relevance of compound **3f** for potential human therapeutic use, we extended the docking study to human α-glucosidase (PDB ID: 3TOP). Consistently, compound **3f** demonstrated a comparable binding affinity to andrographolide, with AutoDock VinaXB-predicted binding energies (*E*_binding_) of –8.4 and –8.2 kcal/mol, respectively. Of particular note, compound **3f** also exhibited a higher binding affinity than the well-known inhibitor acarbose for both human and yeast α-glucosidase enzymes, with binding energies of –8.1 kcal/mol and –8.2 kcal/mol, respectively (Table 1). These results suggest that compound **3f** retains strong binding potential across both yeast and human α-glucosidase, indicating its promise as a hit compound for developing as an α-glucosidase inhibitor with potent antioxidant activity. Analysis of the best-docked poses revealed that compound **3f** formed a greater number of interactions with α-glucosidase and showed a similar pattern in both yeast and human strains, which was primarily driven by van der Waals (vdW) forces, followed by hydrophobic interactions (such as π–alkyl, π–π T-shaped interactions, etc.) and hydrogen bonds (H-bond) (S3 Fig). It is important to note that the *E*_binding_ values obtained from conventional molecular docking do not account for protein flexibility and explicit solvation effects during protein–ligand complex formation [43,44]. Therefore, molecular dynamics (MD) simulations were subsequently performed to gain deeper insights into protein–ligand recognition and to estimate binding free energies more accurately, as discussed in the following section.

**Table 1:** Predicted binding energy in kcal/mol of andrographolide, compound 3f, and acarbose against yeast and human α-glucosidase.

Compound	Binding Energy (kcal/mol)	
	Yeast α-glucosidase	Human α-glucosidase
Andrographolide	–9.1	–8.2
3f	–8.9	–8.4
Acarbose	–8.1	–8.2

#### Drug-likeness.

To evaluate the drug-likeness of compound **3f**, key physicochemical parameters were assessed, including molecular weight (MWT), the number of hydrogen bond donors (HBD) and acceptors (HBA), and the Moriguchi Log P (MLogP). These properties were examined per Lipinski’s Rule, a widely used guideline for predicting oral bioavailability and overall drug-likeness. As shown in Table 2, the predicted values for both andrographolide and compound **3f** generally fall within the acceptable thresholds defined by the rule: HBD ≤ 5, HBA ≤ 10, MLogP ≤ 4.15, and MWT ≤ 500. However, compound **3f** did exhibit a molecular weight exceeding 500 Da. Despite this single exception, compound **3f** is still considered to retain the overall criteria, as minor deviations are permissible within the framework of Lipinski’s rule. These findings suggest that compound **3f** demonstrates favorable drug-like properties, indicating its potential as a promising candidate for further development as a novel α-glucosidase inhibitor. Nevertheless, while these assessments are useful for initial screening, they were limited to *in silico* predictions of drug-likeness. Other crucial aspects of drug development, such as metabolic stability, toxicity, bioavailability, and *in vivo* efficacy, also need to be considered. Therefore, future studies are necessary to validate the therapeutic potential of compound **3f** and address any potential liabilities arising from these predictions.

**Table 2:** Predicted physicochemical properties of andrographolide and compound 3f relevant to Lipinski’s Rule, including molecular weight (MWT), hydrogen bond donors (HBD), hydrogen bond acceptors (HBA), and Moriguchi Log P (MLogP).

Compound	Physicochemical properties				Drug-likeness
	MWT	HBD	HBA	MLogP	Lipinski’s rule
Andrographolide	350.21	3	5	1.98	Yes, 0 violation
3f	670.27	1	7	3.46	Yes, 1 violation

#### Interaction between α-glucosidase and compound 3f via Surface Plasmon Resonance (SPR).

**Fig 9:**
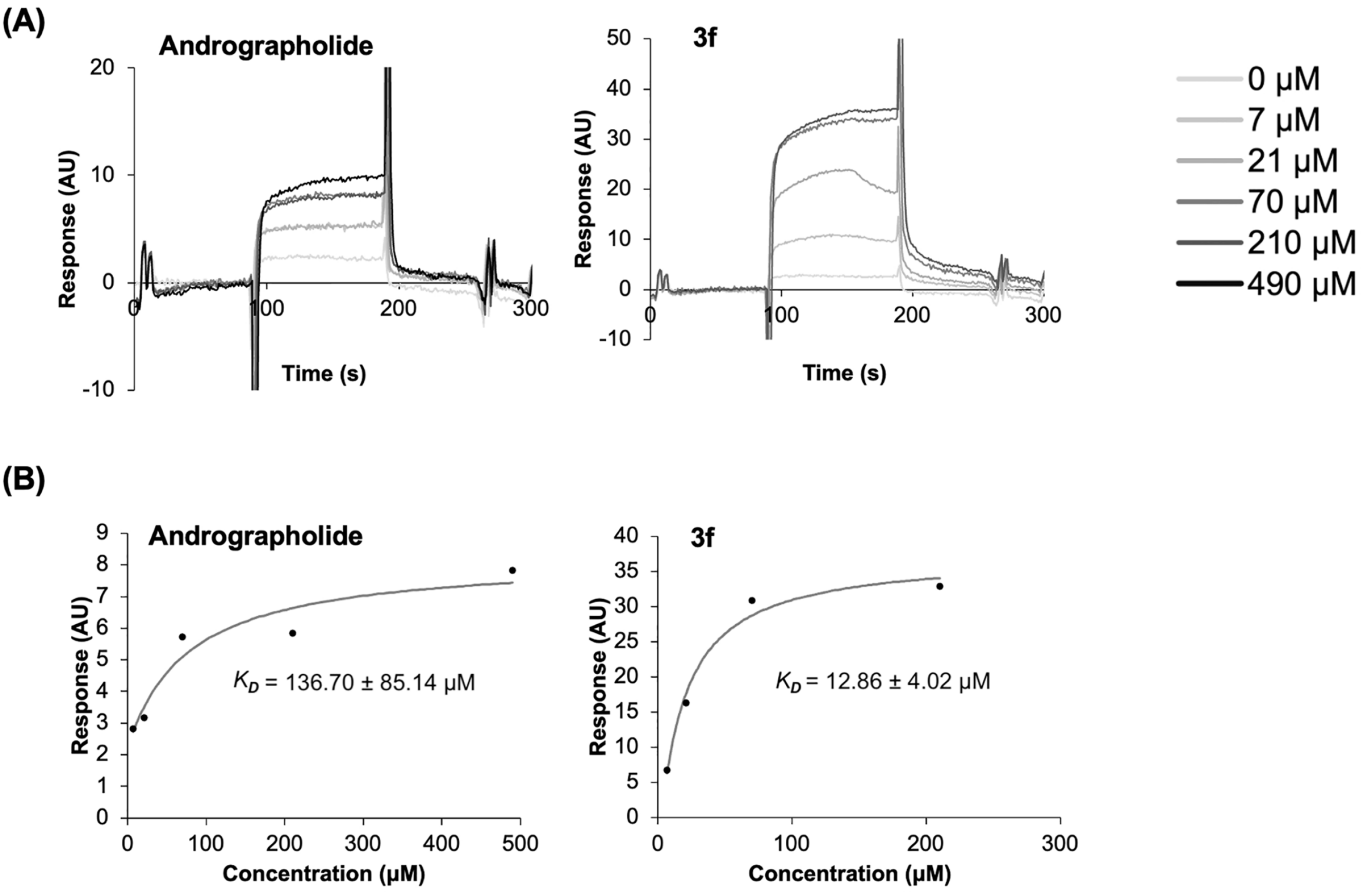
SPR concentration-signal graph. The affinity was determined by steady-state affinity model. (A) Sensorgram of the compounds. (B) Determination of dissociation constant based on the data from the sensorgrams.

To verify the interaction between α-glucosidase and the candidate compounds, we performed surface plasmon resonance, a biophysical technique that measures the affinity of an interaction between an analyte and an immobilized target. Yeast α-glucosidase is commonly used as a screening model to assess the inhibitory effects of compounds on α-glucosidase due to the ease of purification compared to mammalian sources, which pose challenges in obtaining purified α-glucosidase [7,45,46]. The α-glucosidase was attached to an SPR chip, and the compounds were sequentially injected onto this immobilized surface. The interaction between each compound and the protein was reported as a binding response on a sensorgram, which displayed responses during both the association (indicated by increasing response) and dissociation (indicated by decreasing response) phases between the protein and the compound (Fig 9A). The sensorgrams of the compounds injected at various concentrations indicated that andrographolide and compound **3f** bind to the immobilized α-glucosidase in a dose-dependent manner (Fig 9A).

From the sensorgrams obtained from both compounds, the association (*k*_on_) and dissociation rate (*k*_off_) constants were beyond the detection limit of the SPR system and therefore could not be accurately determined. However, the binding affinity between α-glucosidase and the compounds was evaluated by measuring the steady-state binding responses from the sensorgrams and plotting these values against the corresponding analyte concentrations, allowing for the determination of the dissociation constant (*K*_D_) (Fig 9B). From the steady-state analyses, compound **3f** exhibited approximately 10-fold stronger affinity (*K*_D_ = 12.86 ± 4.02 μM than andrographolide (*K*_D_ = 136.70 ± 85.14 μM). The stronger binding affinity of compound **3f** is consistent with its improved end-point binding free energy compared to andrographolide.

#### α-glucosidase inhibitory activity.

**Fig 10:**
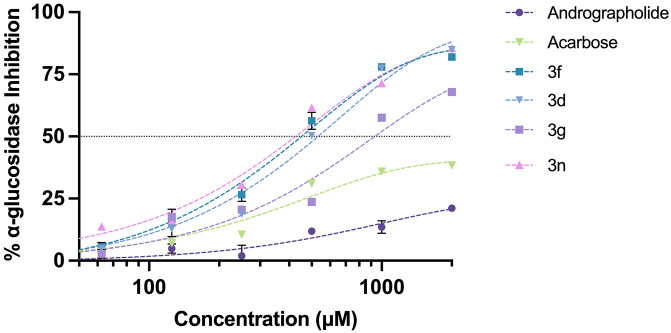
α-glucosidase inhibition curves of compounds 3f, 3d, 3g, 3n, andrographolide, and acarbose. Data are presented as mean ± SD (n = 3).

An *in vitro* α-glucosidase inhibition assay is typically used to assess the ability of compounds to inhibit the α-glucosidase enzyme, which plays a significant role in carbohydrate digestion and influences postprandial blood glucose levels in diabetes. To investigate the potential correlation between α-glucosidase inhibition and the antioxidant activity. The previously identified hit compound **3f** was tested for its inhibitory activity *in vitro*. Additionally, compounds **3d**, **3g**, and **3n**, showing potent antioxidant properties, were also tested to explore this relationship within the context of anti-diabetic drug development. The concentration-response curves of the α-glucosidase inhibitor were shown in Fig 10. At a concentration range up to 2000 μM, compounds **3f**, **3d**, **3g**, and **3n** demonstrated a half-maximal inhibitory concentration (IC_50_) of approximately 411.1 ± 39.8, 527.2 ± 11.7, 929.6 ± 24.3, and 429.7 ± 3.1 μM, respectively (Table 3). By contrast, neither andrographolide nor acarbose achieved an IC_50_ within the tested concentration range; both showed only modest inhibition at the maximum tested concentration of 2000 μM, with inhibition rates of 21.14 ± 1.30% and 38.39 ± 0.96%, respectively (Table 3). These findings demonstrate that compounds with strong antioxidant activity possess markedly stronger inhibitory activity compared to andrographolide and acarbose under the tested conditions (*p* < 0.001). Compound **3f** still appears to be the most promising candidate, supporting a potential link between antioxidant and α-glucosidase inhibitory activities. However, further evaluation against mammalian α-glucosidase enzyme assays and *in vivo* models is necessary to validate therapeutic potential for T2DM by delaying starch breakdown and reducing postprandial hyperglycemia.

**Table 3:** IC_50_ values for the α-glucosidase inhibitory activity of andrographolide, compounds 3f, 3d, 3g, and 3n, and acarbose.

Compound	IC_50_ (μM)
**Andrographolide**	> 2000 (21.14 ± 1.30% Inhibition at 2000 μM)
**3f**	441.1 ± 39.8 ***
**3d**	527.2 ± 11.7 ***^,#^
**3g**	929.6 ± 24.3 ***^,###^
**3n**	429.7 ± 3.1 ***
**Acarbose**	> 2000 (38.39 ± 0.96% Inhibition at 2000 μM)

Data were represented as mean ± SD.

*** represents statistical significance compared to andrographolide and acarbose

(*p* < 0.001). ^#,###^ represents statistical significance compared to compound **3f**. (*p* < 0.05 and 0.001, respectively).

### Molecular dynamics simulations

#### Structural dynamics and binding recognition.

In this final section, MD simulations were performed to investigate the binding affinity and interactions in the dynamic and aqueous surroundings between compound **3f** and human α-glucosidase, compared to a parental andrographolide. This computational approach, widely utilized in biomolecular research, enables the analysis of protein-ligand binding recognition and susceptibility [47–49] in a near-physiological environment. As mentioned in the previous section, while this study primarily focused on validation and evaluation using a yeast enzyme model, the goal is to translate these findings to human applications as potential anti-diabetic agents. Accordingly, the MD simulations provided insights into structural dynamics, binding recognition, and thermodynamic free energy of binding, offering a preliminary understanding of its relevance in a human enzyme context.

**Fig 11:**
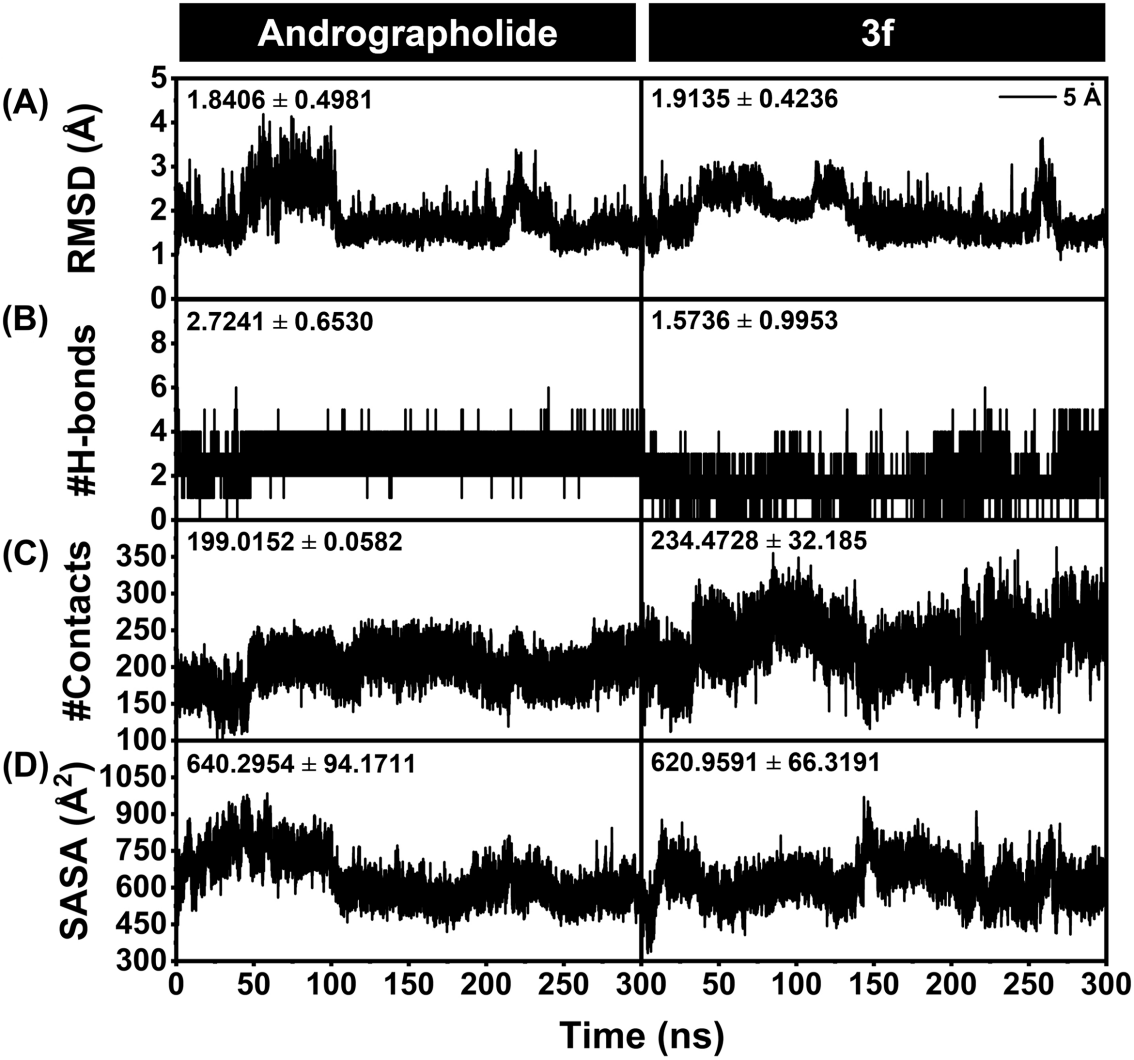
Analysis of structural stability and ligand recognition between α-glucosidase and 3f compared to andrographolide. (A) root-mean square deviation (RMSD), (B) intermolecular H-bond, (C) numbers of all-atom contacts, and (D) solvent-accessible surface area (SASA).

To assess the binding stability, the root-mean-square deviation (RMSD) of backbone amino acids within 5 Å from a ligand was analyzed. As illustrated in Fig 11, both andrographolide and compound **3f** showed a similar trend of RMSD, which deviated at around the first 100 ns and became less deviated when the simulation time increased. However, the RMSD exhibited a slight fluctuation at around 220–240 ns (α-glucosidase-andrographolide) and 250 ns (α-glucosidase – compound **3f** complex), yet became stable afterward, suggesting that both compounds could form a stable complex with human α-glucosidase and at 250–300 ns might reach their binding equilibrium. In addition, we computed the number of hydrogen bonds (H-bond), which is one of the interactions frequently involved in drug binding [50,51]. It was found that andrographolide maintained 2–4 H-bonds with α-glucosidase. Similarly, compound **3f** could also form approximately 2–4 H-bonds, especially during 260–300 ns, the H-bonds were found to be stable at four bonds (Fig 11B). Even though compound **3f** could interact with α-glucosidase via H-bond as like andrographolide, this type of interaction might not be dominantly responsible for its binding, elaborating the discussion in terms of energetic contribution in the following section. Apart from RMSD and H-bond analyses, it is worth counting the all-atom contacts to imply the number of atoms participating in the ligand recognition. Herein, the 5 Å contacts were calculated, and we found that compound **3f** exhibited gradually higher numbers of contacts (Fig 11C), with an average of 234.47 ± 32.18 atoms than andrographolide (average of 199.02 ± 24.15). This might imply that compound **3f** could be likely to have greater numbers of interactions and higher binding affinity to α-glucosidase when compared to andrographolide. In accordance with all-atom contacts, compound **3f** showed a lower solvent-accessible surface area (SASA, Fig 11D) than andrographolide (average values of 620.96 ± 66.32 Å² and 640.29 ± 94.17 Å², respectively). A reduced SASA value suggests a well-buried to the cleft of the binding site, with the possibility to gain direct interactions with surrounding amino-acid residues. Overall, MD simulations suggest that compound **3f** possesses a great binding affinity and stability with α-glucosidase and could have higher non-covalent interactions than andrographolide.

#### End-point free energy of binding.

To assess the ligand binding strength, the last 50-ns MD snapshots (250−300 ns) were used to calculate the end-point free energy of binding (${\Delta G}_{bind}$) by MMPB/GBSA methods. As listed in Table 4, both approaches revealed that compound **3f** could have a slightly lower ${\Delta G}_{bind}$than its parental andrographolide, indicating that it could bind more tightly in the aqueous environment. As observed in the previous section, the improved ${\Delta G}_{bind}$ of compound **3f** might be from the higher number of all-atom contacts and lower SASA values, which correspond to the lower ΔE_vdW_ and ΔG_nonpolar_ in MMPB/GBSA calculations. Specifically, the distinct mode of binding between andrographolide and compound **3f** was observed. Compound **3f** recognized the protein through both electrostatic (ΔE_electrostatic_) and van der Waals (ΔE_vdW_) interaction energies (ΔE = −36.95 ± 0.27 and −43.57 ± 0.18 kcal/mol, respectively), whilst andrographolide formed a complex by the dominant ΔE_electrostatic_, which is approximately 1.6-fold lower than ΔE_vdW_. This finding also suggests that structural modification of compound **3f** enhances the van der Waals interaction energy and is favorable for being buried in the solvation system. It is worth noting that the trend binding affinity prediction from the MD snapshots aligns well with binding energy obtained from molecular docking and experimental *K*_D_ values from the SPR method, as discussed earlier and showed the same trend where *ΔG*_*bind*_ value of compound **3f** was better than andrographolide and acarbose (S4 Table).

**Table 4:** Energy components and *ΔG*_*bind*_ values (kcal/mol) ± SD from the last 50-ns MD snapshots of andrographolide and compound 3f in complex with human α-glucosidase estimated by MMPB/GBSA methods.

Energy components	Andrographolide	3f
*Gas phase (MM)*		
ΔE_electrostatic_	−47.6297 ± 5.8449	−36.9475 ± 6.0290
ΔE_vdW_	−29.6008 ± 3.1814	−43.5700 ± 4.0595
ΔE_gas_	−77.2305 ± 5.5070	−80.5175 ± 7.1473
*Solvation (GBSA)*		
ΔG_polar_	57.5356 ± 4.9850	59.0431 ± 5.7619
ΔG_nonpolar_	−4.4425 ± 0.2492	−5.9563 ± 0.4359
*Solvation (PBSA)*		
ΔG_polar_	56.4987 ± 4.7346	61.4853 ± 7.9140
ΔG_nonpolar_	−5.0899 ± 0.2083	−7.3655 ± 0.4210
*ΔG*_*bind (MM/GBSA)*_	−24.1373 ± 2.7381	−27.4307 ± 3.6713
*ΔG*_*bind (MM/PBSA)*_	−25.8217 ± 2.9901	−26.3977 ± 5.4994

Data were represented as mean ± SD.

#### Hot-spot amino acids for ligand binding.

**Fig 12:**
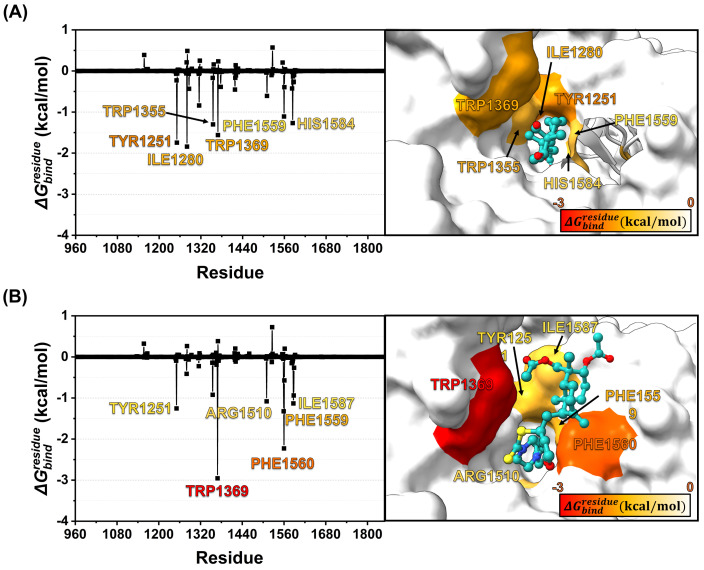
ΔGbindresidue ${\Delta 𝐆}_{bind}^{residue}$ (kcal/mol) of the crucial residue for (A) andrographolide and (B) compound 3f binding with human α-glucosidase.

To gain insights into ligand binding modes, the key amino acids, playing a dominant role in recognition, were identified by the predicted decomposition free energy of binding (${\Delta G}_{bind}^{residue}$). Note that only residues exhibiting ${\Delta G}_{bind}^{residue}$ <−1.00 kcal/mol were considered “hotspots”. As illustrated in Fig 12, key residues involved in andrographolide binding include Tyr1251, Ile1280, Trp1355, Trp1369, Phe1559, and His1584 (Fig 12A), while the critical binding residues for compound **3f** are Tyr1251, Trp1369, Arg1510, Phe1559, Phe1560, and Ile1587 (Fig 12B). Interestingly, we identified similar residues between andrographolide and compound **3f**, including Tyr1251, Trp1369, and Phe1559. This suggests that both the parent compound and its analog likely recognize the same amino acids, contributing to the stability of ligand binding upon complex formation. Moreover, Trp1396 and Phe1560 might be critical residues for compound **3f**’s binding. They showed a dominant role in stabilizing the ligand occupation with the lowest ${\Delta G}_{bind}^{residue}$ among all residues observed in both compounds. Almost all identified key residues for compound **3f**’s binding were also observed in the binding of acarbose [27], a well-known α-glucosidase inhibitor (Tyr1251, Trp1369, Arg1510, Phe1559, and Phe1560), suggesting a possibly similar mechanism of action, resulting in an effective inhibitory activity.

## Conclusion

This study evaluated the antioxidant activity and α-glucosidase inhibition of the potential compound **3f** through both *in vitro* and *in silico* analyses. Among the C-12 dithiocarbamate andrographolide analogues and andrographolide, compound **3f** exhibited the most potent antioxidant activity and demonstrated significantly greater α-glucosidase inhibition than the parent compound, andrographolide, and the reference inhibitor, acarbose. SPR analysis further confirmed a strong binding affinity of compound **3f** for α-glucosidase. The MD simulations revealed stable and thermodynamically favorable binding to human α-glucosidase, suggesting its potential translational relevance in a human enzyme context. Furthermore, compound **3f** exhibited physicochemical properties consistent with Lipinski’s Rule of Five, suggesting favorable drug-likeness for further development. While compound **3f** shows promise as a novel α-glucosidase inhibitor, this study has limitations that warrant further investigation. Of particular note, validation was conducted using a yeast α-glucosidase model, and data on efficacy and safety in human systems remain lacking. Future *in vivo* studies, along with pharmacokinetic ADMET analysis, will be essential to assess its translational potential. Thus, the present findings are considered an initial screening step, with additional validation in cellular and animal models needed to confirm *in vivo* relevance to further develop as a potential anti-diabetic therapy.

## Supporting information

S1 Fig *De novo* three-dimensional yeast α-glucosidase protein construction using AlphaFold 3. (A) An amino-acid sequence of yeast α-glucosidase obtained from Uniprot (MAL32 gene, ID: P38158). (B) The top-ranked AlphaFold model of yeast α-glucosidase. (C) A residue-wise alignment with predicted local distance error. High-confidence residues are colored blue, and lower confidence in yellow, orange, and red. (D) Ramachandran plot of yeast α-glucosidase structure, estimating 98.454% favored regions.
(DOCX)

S2 Fig The top-ranked druggability site of yeast α-glucosidase identified by Fpocket. This site was used as a docking center for molecular docking.
(DOCX)

S3 Fig 2D interaction diagrams and binding energies of yeast and human α-glucosidase in complex with compounds. (A) andrographolide, (B) compound **3f**. The interactions were analyzed based on the best docking poses, as determined by AutoDock VinaXB scores, and visualized using BIOVIA Discovery Studio Visualizer 2021. Different colors represent distinct types of intermolecular interactions. Note that polar hydrogen was automatically shown only when it had interactions with amino acids.
(DOCX)

S4 Fig Alignment of original and redocked conformations of co-crystallized ligand (acarbose) with the docking parameters. The alignment and visualization were done using the ChimeraX 1.8 program.
(DOCX)

S5 Fig SPR analysis of acarbose against immobilised α-glucosidase. (A) sensorgram, (B) steady-state affinity analysis.
(DOCX)

S6 Fig The raw sensorgrams of the compound interacting with the reference (no ligand) and the sample (immobilised ligand) flow cells. (A) Raw sensorgram for compound **3f**-reference, (B) Raw sensorgram for compound **3f**-immobilised, (C) Raw sensorgram for andrographolide-reference, (D) Raw sensorgram for andrographolide-immobilised.
(DOCX)

S1 Table Antioxidant activity of the screening compounds at a concentration of 500 μM: DPPH scavenging activity. (DOCX)

S2 Table Antioxidant activity of the screening compounds at a concentration of 500 μM: Ferric reducing antioxidant power (FRAP). (DOCX)

S3 Table α-glucosidase inhibitory activity (%) of 3f, 3g, 3d, 3n, andrographolide, and acarbose at a concentration range up to 2000 μM. (DOCX)

S4 Table Energy components and *ΔG*_*bind*_ values (kcal/mol) ± SD from the last 50-ns MD snapshots of acarbose in complex with human α-glucosidase estimated by MMPB/GBSA methods. (DOCX)
