# Supplementary material for: Discovery of C-12 dithiocarbamate andrographolide analogue as a novel antioxidant and α-glucosidase inhibitors: In vitro and in silico studies
Source: PLoS One. 2025 Oct 22;20(10):e0334026. doi: 10.1371/journal.pone.0334026 (PMC12543186; doi:10.1371/journal.pone.0334026)
Supplement: S1 Fig — (A) An amino-acid sequence of yeast α-glucosidase obtained from Uniprot (MAL32 gene, ID: P38158). (B) The top-ranked AlphaFold model of yeast α-glucosidase. (C) A residue-wise alignment with predicted local distance error. High-confidence residues are colored blue, and lower confidence in yellow, orange, and red. (D) Ramachandran plot of yeast α-glucosidase structure, estimating 98.454% favored regions. (DOCX) [file pone.0334026.s001.docx]

**Supporting information**

**
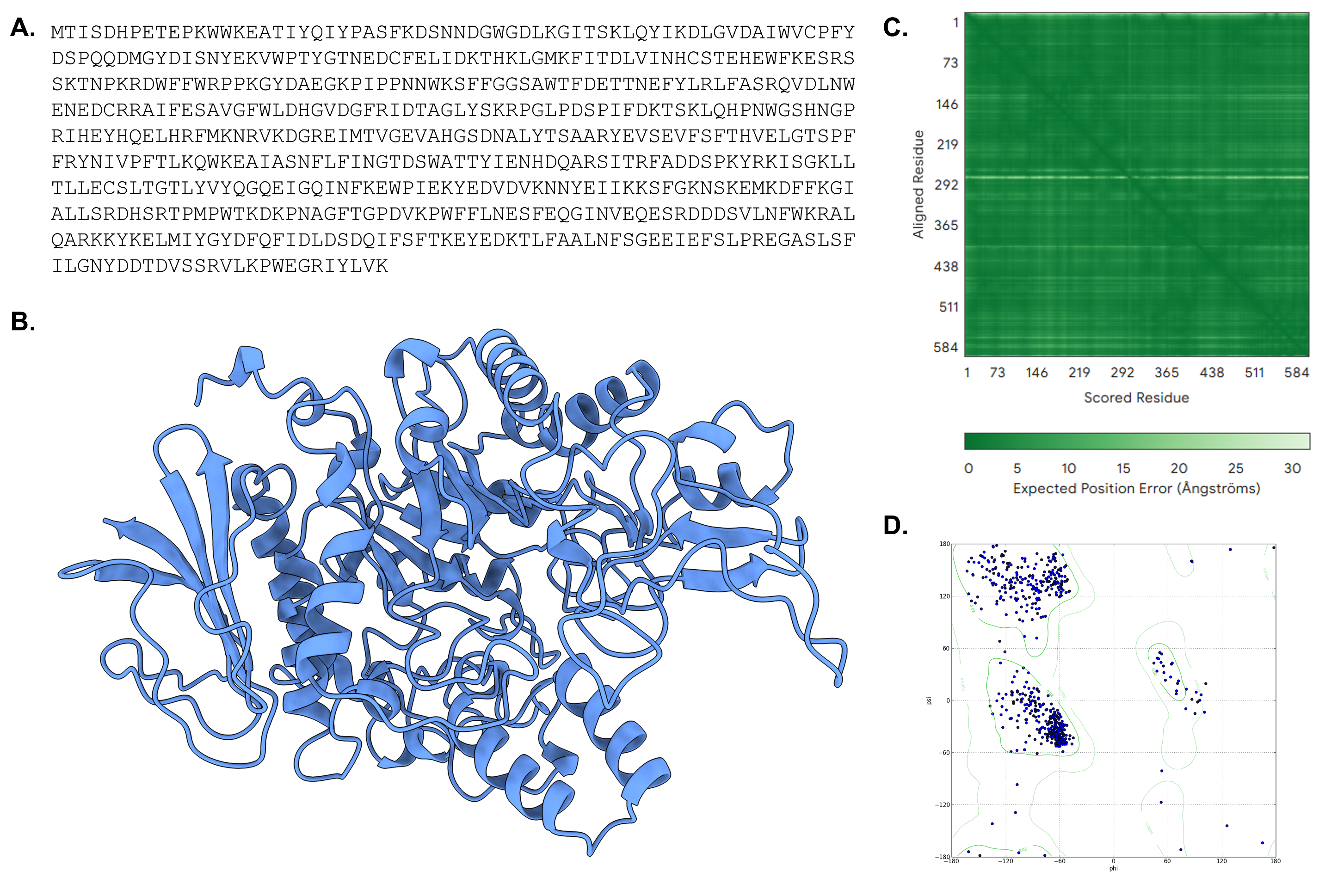
**

**S1 Fig: *De novo* three-dimensional yeast α-glucosidase protein construction using AlphaFold 3.** (A) An amino-acid sequence of yeast α-glucosidase obtained from Uniprot (MAL32 gene, ID: P38158). (B) The top-ranked AlphaFold model of yeast α-glucosidase. (C) A residue-wise alignment with predicted local distance error. High-confidence residues are colored blue, and lower confidence in yellow, orange, and red. (D) Ramachandran plot of yeast α-glucosidase structure, estimating 98.454% favored regions using Ramplot (available in <https://www.ramplot.in>, accessed 16 September 2025).
