# Supplementary material for: Discovery of C-12 dithiocarbamate andrographolide analogue as a novel antioxidant and α-glucosidase inhibitors: In vitro and in silico studies
Source: PLoS One. 2025 Oct 22;20(10):e0334026. doi: 10.1371/journal.pone.0334026 (PMC12543186; doi:10.1371/journal.pone.0334026)
Supplement: S2 Fig — This site was used as a docking center for molecular docking. (DOCX) [file pone.0334026.s002.docx]

**Supporting information**

**
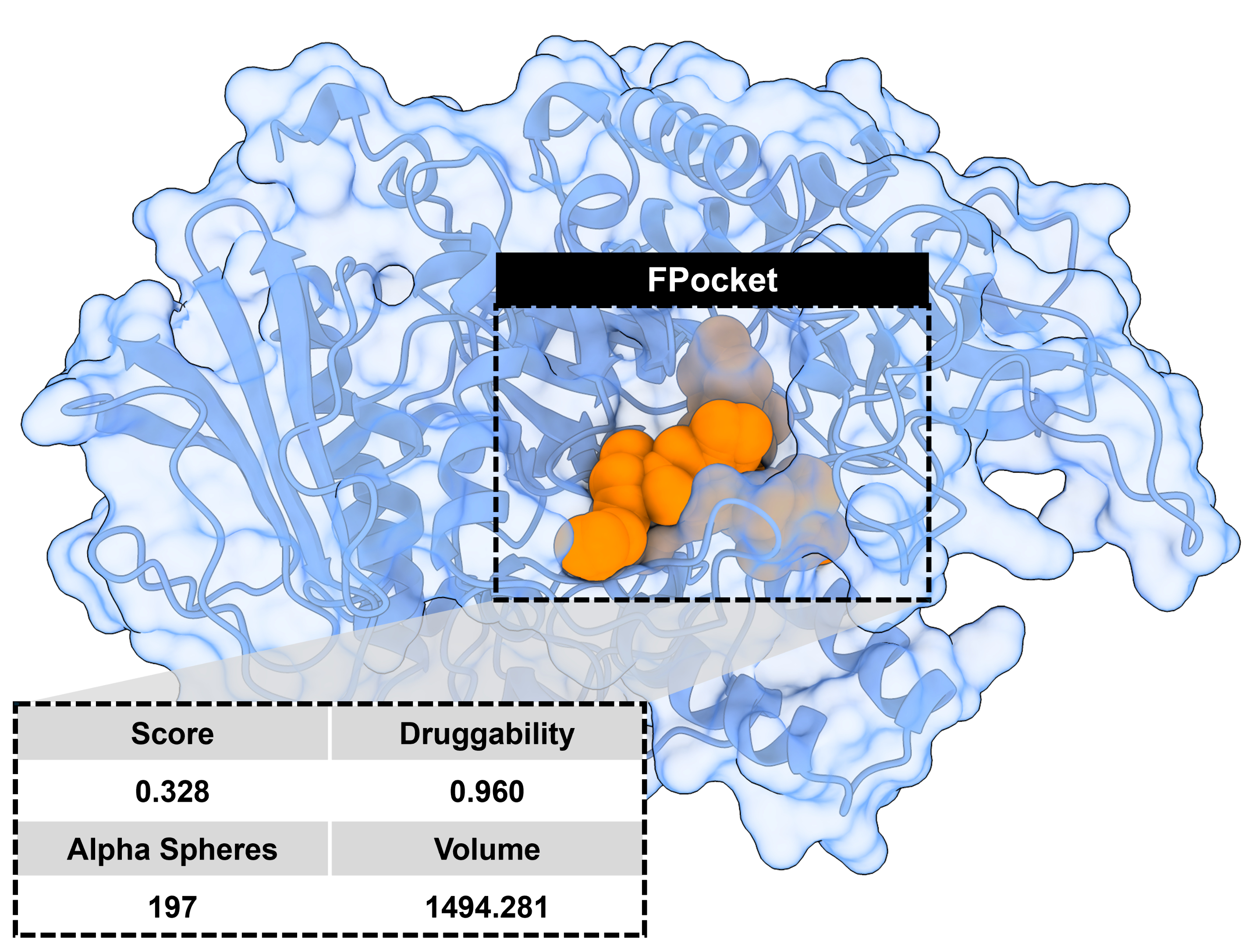
**

**S2 Fig: The top-ranked druggability site of yeast α-glucosidase identified by Fpocket. This site was used as a docking center for molecular docking.**
