# Supplementary material for: Discovery of C-12 dithiocarbamate andrographolide analogue as a novel antioxidant and α-glucosidase inhibitors: In vitro and in silico studies
Source: PLoS One. 2025 Oct 22;20(10):e0334026. doi: 10.1371/journal.pone.0334026 (PMC12543186; doi:10.1371/journal.pone.0334026)
Supplement: S3 Fig — (A) andrographolide, (B) compound 3f. The interactions were analyzed based on the best docking poses, as determined by AutoDock VinaXB scores, and visualized using BIOVIA Discovery Studio Visualizer 2021. Different colors represent distinct types of intermolecular interactions. Note that polar hydrogen was automatically shown only when it had interactions with amino acids. (DOCX) [file pone.0334026.s003.docx]

**Supporting information**

**
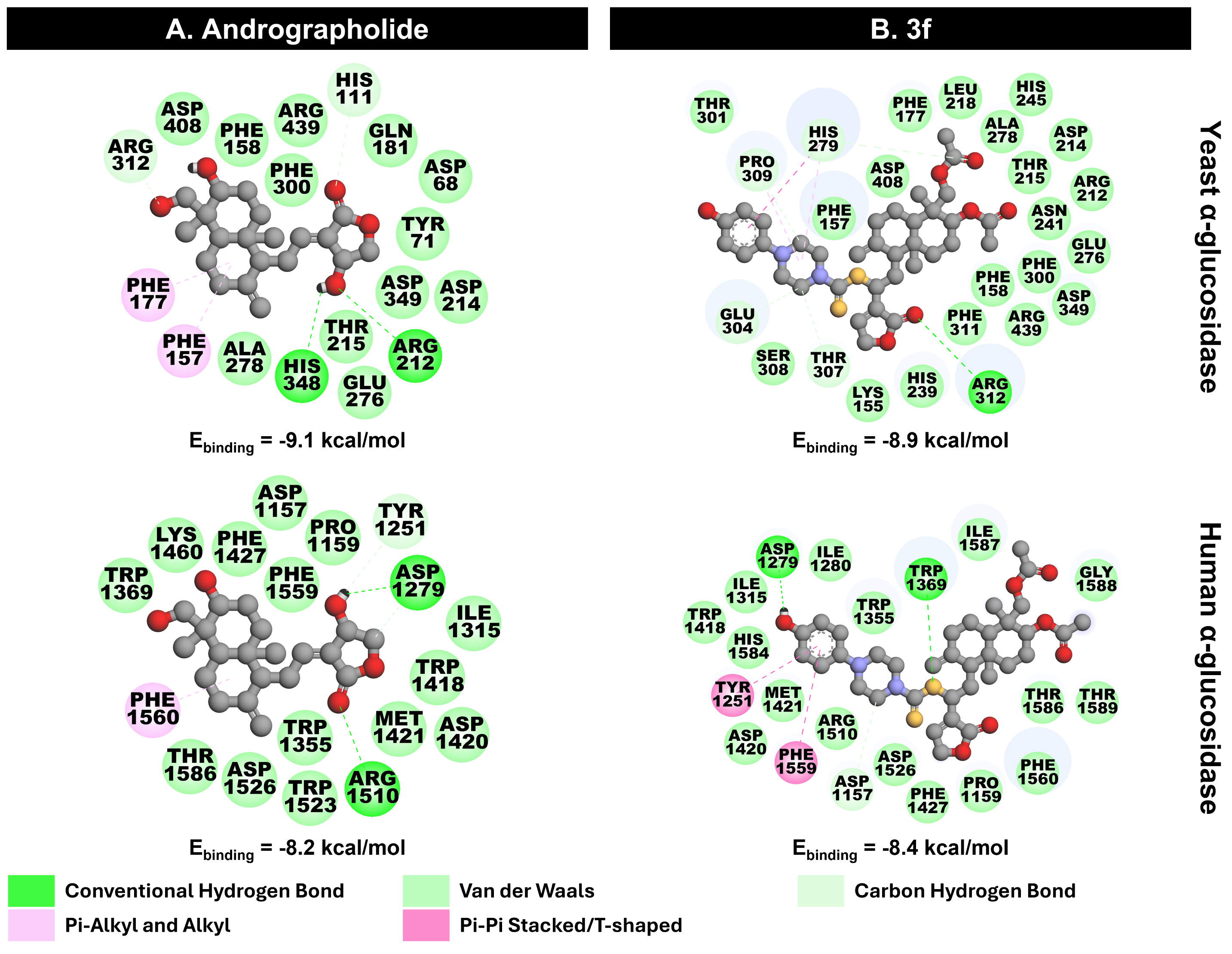
**

**S3 Fig: 2D interaction diagrams and binding energies of yeast and human α-glucosidase in complex with compounds.** (A) andrographolide, (B) compound 3f. The interactions were analyzed based on the best docking poses, as determined by AutoDock VinaXB scores, and visualized using BIOVIA Discovery Studio Visualizer 2021. Different colors represent distinct types of intermolecular interactions. Note that polar hydrogen was automatically shown only when it had interactions with amino acids.
