# Supplementary material for: Discovery of C-12 dithiocarbamate andrographolide analogue as a novel antioxidant and α-glucosidase inhibitors: In vitro and in silico studies
Source: PLoS One. 2025 Oct 22;20(10):e0334026. doi: 10.1371/journal.pone.0334026 (PMC12543186; doi:10.1371/journal.pone.0334026)
Supplement: S4 Fig — The alignment and visualization were done using the ChimeraX 1.8 program. (DOCX) [file pone.0334026.s004.docx]

**Supporting information**

**
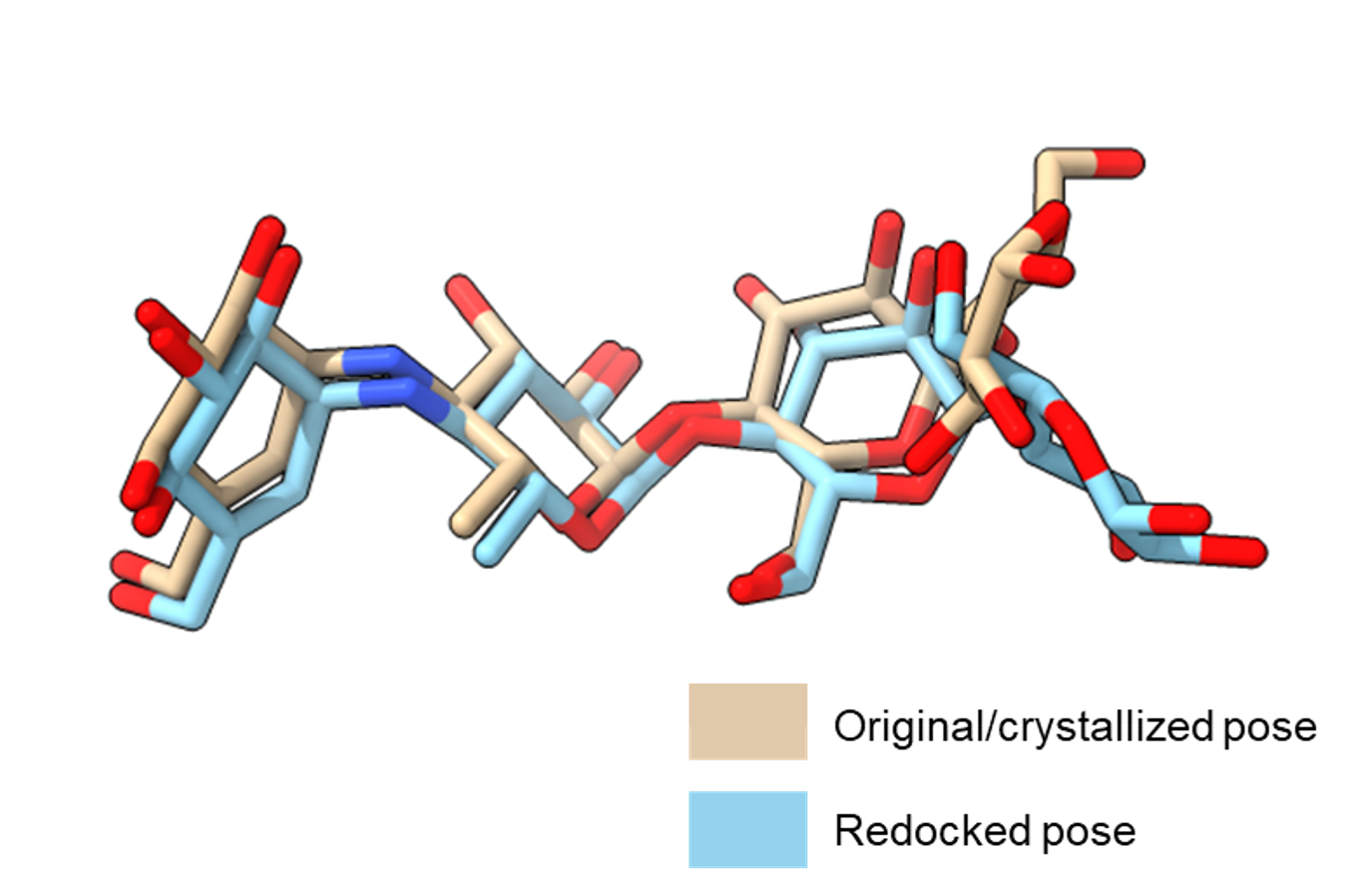
**

**S4 Fig.** **Alignment of original and redocked conformations of co-crystallized ligand (acarbose) with the docking parameters.** The alignment and visualization were done using the ChimeraX 1.8 program.
