# Supplementary material for: Discovery of C-12 dithiocarbamate andrographolide analogue as a novel antioxidant and α-glucosidase inhibitors: In vitro and in silico studies
Source: PLoS One. 2025 Oct 22;20(10):e0334026. doi: 10.1371/journal.pone.0334026 (PMC12543186; doi:10.1371/journal.pone.0334026)
Supplement: S5 Fig — (A) sensorgram, (B) steady-state affinity analysis. (DOCX) [file pone.0334026.s005.docx]

**Supporting information**

**
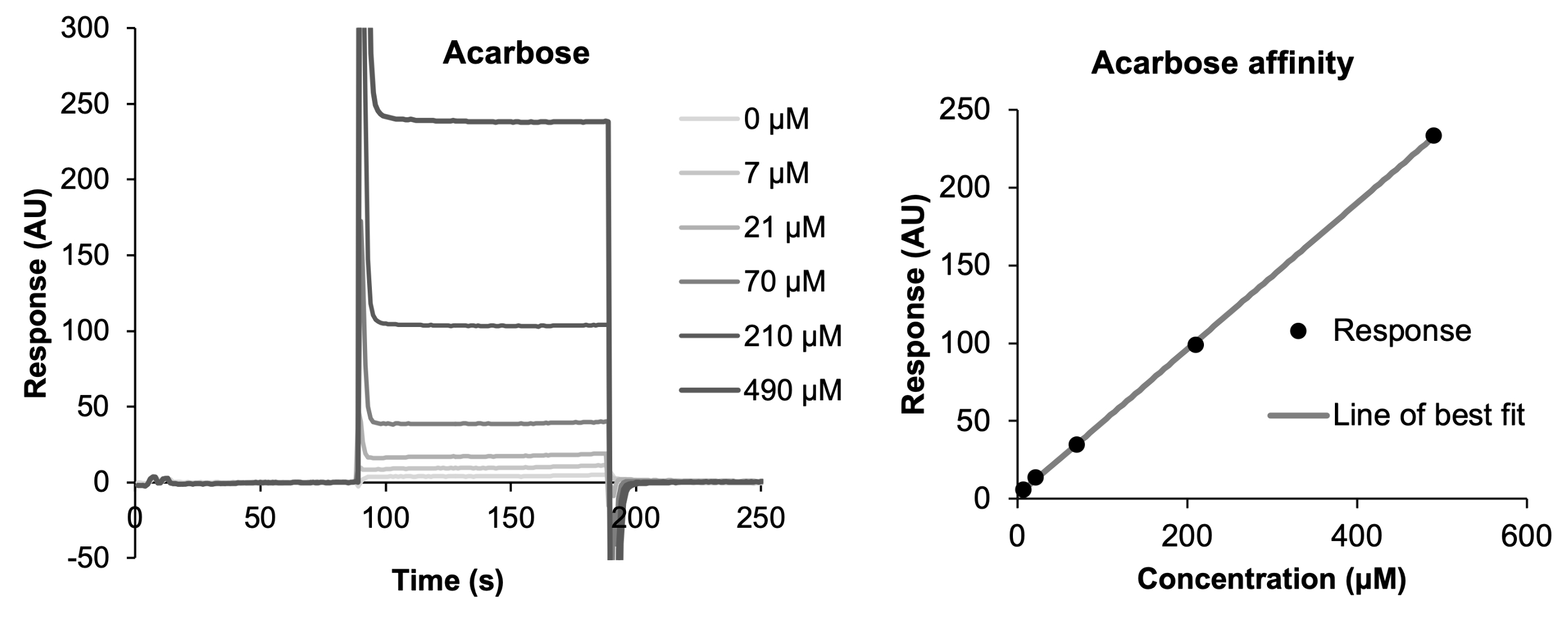
**

**S5 Fig. SPR analysis of acarbose against immobilised α-glucosidase.**(A) sensorgram, (B) steady-state affinity analysis.
