# Supplementary material for: Discovery of C-12 dithiocarbamate andrographolide analogue as a novel antioxidant and α-glucosidase inhibitors: In vitro and in silico studies
Source: PLoS One. 2025 Oct 22;20(10):e0334026. doi: 10.1371/journal.pone.0334026 (PMC12543186; doi:10.1371/journal.pone.0334026)
Supplement: S6 Fig — (A) Raw sensorgram for compound 3f-reference, (B) Raw sensorgram for compound 3f-immobilised, (C) Raw sensorgram for andrographolide-reference, (D) Raw sensorgram for andrographolide-immobilised. (DOCX) [file pone.0334026.s006.docx]

**Supporting information**


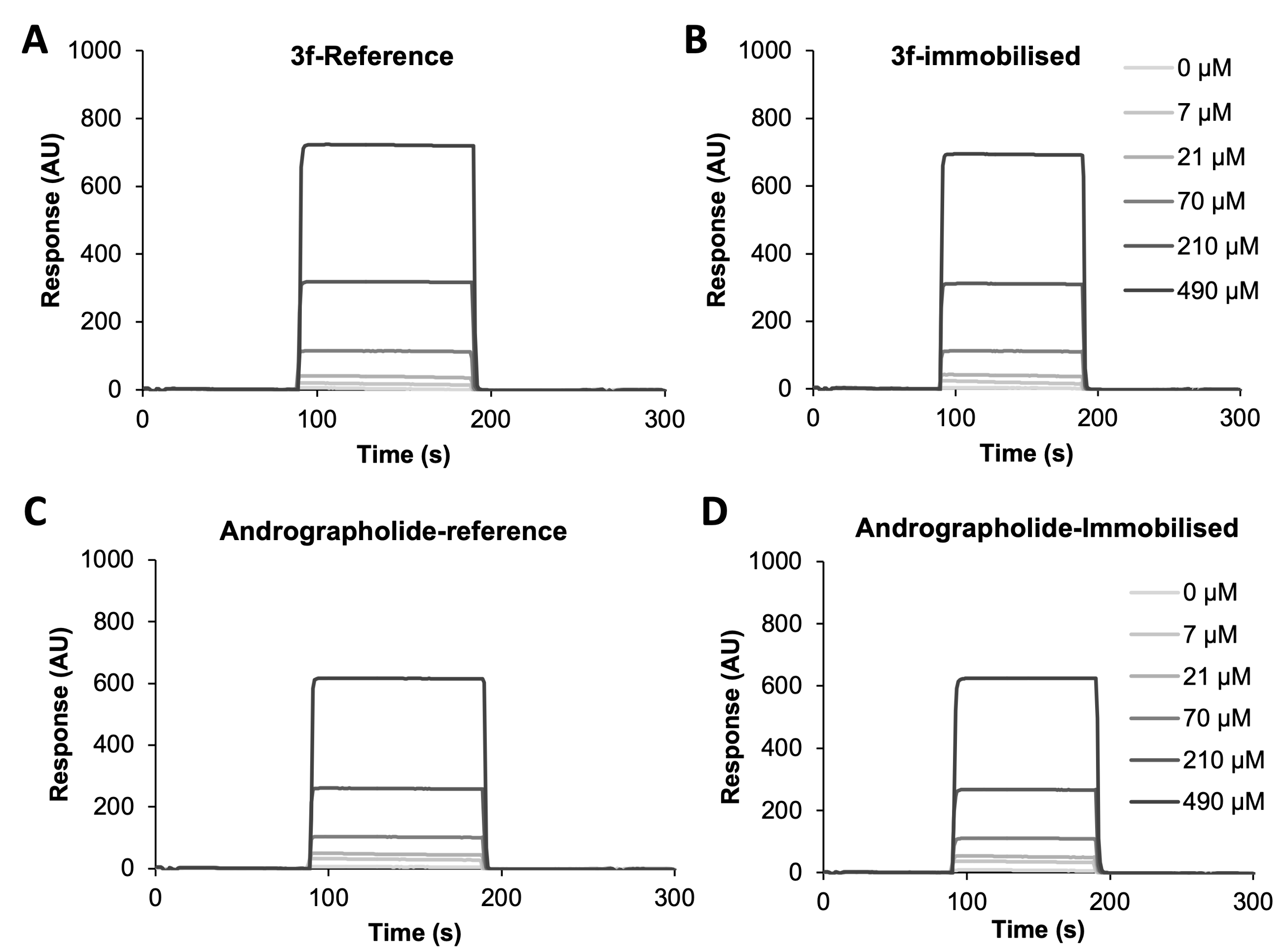


**S6 Fig. The raw sensorgrams of the compound interacting with the reference (no ligand) and the sample (immobilised ligand) flow cells.**(A) Raw sensorgram for compound 3f-reference, (B) Raw sensorgram for compound 3f-immobilised, (C) Raw sensorgram for andrographolide-reference, (D) Raw sensorgram for andrographolide-immobilised.
