# Supplementary material for: Discovery of C-12 dithiocarbamate andrographolide analogue as a novel antioxidant and α-glucosidase inhibitors: In vitro and in silico studies
Source: PLoS One. 2025 Oct 22;20(10):e0334026. doi: 10.1371/journal.pone.0334026 (PMC12543186; doi:10.1371/journal.pone.0334026)
Supplement: S1 Table — (DOCX) [file pone.0334026.s007.docx]

**Supporting information**

**S1 Table.** **Antioxidant activity of the screening compounds at a concentration of 500 μM: DPPH scavenging activity**

| **Compound** | **% Inhibition** | | | **Mean** | **SD** |
| --- | --- | --- | --- | --- | --- |
| **Crude*** | 31.3760 | 31.4925 | 31.7367 | **31.5351** | **0.1841** |
| **Andro.** | 17.9296 | 11.6610 | 13.8517 | **14.4808** | **3.1813** |
| **3a** | 21.3677 | 17.7208 | 20.4142 | **19.8343** | **1.8914** |
| **3b** | 19.6817 | 16.7507 | 20.4510 | **18.9612** | **1.9525** |
| **3c** | 20.4175 | 18.9285 | 22.2305 | **20.5255** | **1.6536** |
| **3d** | 35.2972 | 30.8724 | 35.8894 | **34.0196** | **2.7416** |
| **3e** | 20.2567 | 16.5202 | 19.5473 | **18.7747** | **1.9845** |
| **3f** | 85.7465 | 83.5328 | 84.1364 | **84.4719** | **1.1443** |
| **3g** | 32.2816 | 27.0570 | 28.7195 | **29.3527** | **2.6692** |
| **3h** | 21.7355 | 19.8743 | 19.6112 | **20.4070** | **1.1580** |
| **3i** | 17.2873 | 13.5999 | 15.7714 | **15.5529** | **1.8534** |
| **3j** | 18.1336 | 15.6696 | 15.8311 | **16.5447** | **1.3783** |
| **3k** | 16.1513 | 14.7734 | 14.3884 | **15.1044** | **0.9268** |
| **3l** | 19.9024 | 19.3119 | 19.4020 | **19.5388** | **0.3181** |
| **3m** | 15.2811 | 17.7112 | 16.0139 | **16.3354** | **1.2466** |
| **3n** | 30.2593 | 31.0041 | 29.7576 | **30.3403** | **0.6272** |
| **3o** | 14.5862 | 15.7537 | 13.7522 | **14.6974** | **1.0054** |
| **3p** | 11.3686 | 14.4724 | 10.3569 | **12.0660** | **2.1446** |
| **3q** | 22.9566 | 23.9557 | 20.6979 | **22.5367** | **1.6690** |
| **3r** | 20.2051 | 22.6381 | 18.1179 | **20.3204** | **2.2623** |
| **3s** | 26.0220 | 26.6023 | 23.7382 | **25.4542** | **1.5141** |
| **3t** | 17.7391 | 17.7220 | 16.9880 | **17.4830** | **0.4288** |
| **3u** | 22.7692 | 20.1323 | 19.4749 | **20.7921** | **1.7434** |

* Crude extract: 500 mg/mL
