# Supplementary material for: Discovery of C-12 dithiocarbamate andrographolide analogue as a novel antioxidant and α-glucosidase inhibitors: In vitro and in silico studies
Source: PLoS One. 2025 Oct 22;20(10):e0334026. doi: 10.1371/journal.pone.0334026 (PMC12543186; doi:10.1371/journal.pone.0334026)
Supplement: S2 Table — (DOCX) [file pone.0334026.s008.docx]

**Supporting information**

**S2 Table.** **Antioxidant activity of the screening compounds at a concentration of 500 μM: Ferric reducing antioxidant power (FRAP)**

| **Sample** | **Ascorbic acid equivalent  (AAE, μM)** | | | **Mean** | **SD** |
| --- | --- | --- | --- | --- | --- |
| **Crude*** | 11.3128 | 12.1897 | 13.5250 | **12.3425** | **1.1140** |
| **Andro.** | -1.6499 | -1.0884 | -2.0327 | **-1.6499** | **-1.0884** |
| **3a** | 13.0690 | 22.0807 | 10.0223 | **15.0573** | **6.2703** |
| **3b** | 14.5092 | 20.3495 | 8.4425 | **14.4338** | **5.9539** |
| **3c** | 19.3279 | 25.8103 | 16.4063 | **20.5149** | **4.8131** |
| **3d** | 17.6227 | 23.0391 | 19.2323 | **19.9647** | **2.7815** |
| **3e** | 5.7691 | 11.3705 | 10.5501 | **9.2299** | **3.0251** |
| **3f** | 109.9203 | 87.9587 | 67.0175 | **88.2989** | **21.4534** |
| **3g** | 28.8291 | 32.2227 | 28.6035 | **29.8851** | **2.0276** |
| **3h** | 8.3618 | 10.9510 | 6.5910 | **8.6346** | **2.1928** |
| **3i** | 16.7992 | 5.9930 | 5.4484 | **9.4135** | **6.4019** |
| **3j** | 16.9541 | 20.6599 | 13.7537 | **17.1226** | **3.4562** |
| **3k** | 6.7599 | 3.9881 | 7.1620 | **5.9700** | **1.7281** |
| **3l** | 14.7839 | 13.1288 | 12.1825 | **13.3651** | **1.3167** |
| **3m** | 18.8527 | 8.5660 | 16.7016 | **14.7068** | **5.4257** |
| **3n** | 36.4717 | 34.2845 | 34.4617 | **35.0726** | **1.2149** |
| **3o** | 12.3028 | 13.1651 | 10.8913 | **12.1197** | **1.1479** |
| **3p** | 15.7875 | 17.6761 | 9.9560 | **14.4732** | **4.0244** |
| **3q** | 3.3874 | 4.3767 | 3.1585 | **3.6409** | **0.6475** |
| **3r** | 33.4629 | 38.2989 | 33.1745 | **34.9787** | **2.8789** |
| **3s** | 37.2705 | 34.8073 | 29.3633 | **33.8137** | **4.0462** |
| **3t** | 20.3933 | 20.8001 | 12.2711 | **17.8215** | **4.8110** |
| **3u** | 32.5081 | 19.7249 | 16.1959 | **22.8096** | **8.5824** |

* Crude extract: 500 mg/mL
