# Supplementary material for: Discovery of C-12 dithiocarbamate andrographolide analogue as a novel antioxidant and α-glucosidase inhibitors: In vitro and in silico studies
Source: PLoS One. 2025 Oct 22;20(10):e0334026. doi: 10.1371/journal.pone.0334026 (PMC12543186; doi:10.1371/journal.pone.0334026)
Supplement: S3 Table — (DOCX) [file pone.0334026.s009.docx]

**Supporting information**

**S3 Table.** **α-glucosidase inhibitory activity (%) of 3f, 3g, 3d, 3n, andrographolide, and acarbose at a concentration range up to 2000 μM**

| **Conc. (μM)** | **α-glucosidase inhibition (%)** | | | **Mean** | **SD** |
| --- | --- | --- | --- | --- | --- |
| ***Andrographolide*** | | | | | |
| **0** | 0.000 | 0.000 | 0.000 | 0.0000 | 0.0000 |
| **31.2** | -0.3003 | 1.4718 | -0.9760 | 0.0652 | 1.2642 |
| **62.5** | 2.1915 | 3.0555 | -7.4924 | -0.7485 | 5.8564 |
| **125** | 6.7641 | 3.1890 | 5.6784 | 4.8772 | 1.7958 |
| **250** | -2.9735 | 3.6005 | 5.1626 | 1.9299 | 4.3177 |
| **500** | 13.1282 | 12.0147 | 10.6051 | 11.9160 | 1.2644 |
| **1000** | 17.1182 | 13.4995 | 11.0433 | 13.5523 | 2.5379 |
| **2000** | 22.5085 | 21.0075 | 19.9137 | 21.1432 | 1.3027 |
| ***Acarbose*** | | | | | |
| **0** | 0.0000 | 0.0000 | 0.0000 | 0.0000 | 0.0000 |
| **31.2** | 5.5790 | 5.8899 | 8.5353 | 6.6681 | 1.6246 |
| **62.5** | 3.7426 | 3.6342 | 3.8227 | 3.7332 | 0.0946 |
| **125** | 6.9673 | 7.3445 | 7.0555 | 7.1224 | 0.1974 |
| **250** | 10.5247 | 10.7321 | 10.4749 | 10.5772 | 0.1364 |
| **500** | 31.1674 | 31.0583 | 31.1114 | 31.1123 | 0.0546 |
| **1000** | 36.0384 | 36.7142 | 34.8140 | 35.8555 | 0.9632 |
| **2000** | 37.4977 | 39.4023 | 38.2776 | 38.3925 | 0.9575 |
| ***3f*** | | | | | |
| **0** | 0.0000 | 0.0000 | 0.0000 | 0.0000 | 0.0000 |
| **31.2** | 3.1527 | -1.1981 | -3.4237 | -0.4897 | 3.3450 |
| **62.5** | 5.2436 | 7.4006 | 3.9450 | 5.5297 | 1.7455 |
| **125** | 19.1287 | 17.5649 | 8.9261 | 15.2066 | 5.4950 |
| **250** | 28.2057 | 28.0719 | 23.4599 | 26.5792 | 2.7022 |
| **500** | 59.2842 | 57.0845 | 52.5824 | 56.3170 | 3.4162 |
| **1000** | 78.5779 | 77.9655 | 77.3110 | 77.9514 | 0.6336 |
| **2000** | 81.9490 | 81.9182 | 81.9750 | 81.9474 | 0.0284 |
| **3d** | | | | | |
| **0** | 0.0000 | 0.0000 | 0.0000 | 0.0000 | 0.0000 |
| **31.2** | 4.6851 | 3.4514 | 6.2939 | 4.8101 | 1.4254 |
| **62.5** | 5.0509 | 4.7326 | 5.1063 | 4.9633 | 0.2017 |
| **125** | 13.1626 | 11.8790 | 13.4121 | 12.8179 | 0.8226 |
| **250** | 19.0019 | 17.1693 | 19.9318 | 18.7010 | 1.4056 |
| **500** | 50.9883 | 49.4843 | 50.3225 | 50.2651 | 0.7536 |
| **1000** | 77.9173 | 77.0662 | 77.4647 | 77.4828 | 0.4258 |
| **2000** | 84.9886 | 85.1772 | 84.5070 | 84.8909 | 0.3456 |

**S3 Table.** **α-glucosidase inhibitory activity (%) of 3f, 3g, 3d, 3n, andrographolide, and acarbose at a concentration range up to 2000 μM (cont.)**

| **Conc. (μM)** | **α-glucosidase inhibition (%)** | | | **Mean** | **SD** |
| --- | --- | --- | --- | --- | --- |
| ***3g*** | | | | | |
| **0** | 0.0000 | 0.0000 | 0.0000 | 0.0000 | 0.0000 |
| **31.2** | -2.0532 | -1.7725 | -3.7910 | -2.5389 | 1.0934 |
| **62.5** | 3.1105 | 4.3631 | 0.8940 | 2.7892 | 1.7567 |
| **125** | 18.2295 | 18.2433 | 16.4227 | 17.6319 | 1.0472 |
| **250** | 19.8782 | 21.3223 | 20.4403 | 20.5469 | 0.7280 |
| **500** | 24.6136 | 23.9352 | 22.4012 | 23.6500 | 1.1335 |
| **1000** | 58.2403 | 58.0215 | 56.4434 | 57.5684 | 0.9804 |
| **2000** | 68.6727 | 68.0047 | 67.0550 | 67.9108 | 0.8129 |
| ***3n*** | | | | | |
| **0** | 0.0000 | 0.0000 | 0.0000 | 0.0000 | 0.0000 |
| **31.2** | 5.9921 | 8.2939 | 8.6675 | 7.6512 | 1.4489 |
| **62.5** | 12.2472 | 14.0004 | 14.8499 | 13.6992 | 1.3272 |
| **125** | 14.4158 | 17.2043 | 16.6717 | 16.0973 | 1.4803 |
| **250** | 30.7924 | 29.5661 | 31.0657 | 30.4747 | 0.7987 |
| **500** | 61.2808 | 61.7093 | 61.4416 | 61.4772 | 0.2165 |
| **1000** | 71.2224 | 71.1313 | 71.6695 | 71.3411 | 0.2881 |
| **2000** | 85.0717 | 85.9704 | 85.4613 | 85.5011 | 0.4507 |
