# Supplementary material for: Discovery of C-12 dithiocarbamate andrographolide analogue as a novel antioxidant and α-glucosidase inhibitors: In vitro and in silico studies
Source: PLoS One. 2025 Oct 22;20(10):e0334026. doi: 10.1371/journal.pone.0334026 (PMC12543186; doi:10.1371/journal.pone.0334026)
Supplement: S4 Table — (DOCX) [file pone.0334026.s010.docx]

**Supporting information**

**S4 Table.** Energy components and *ΔG_bind_* values (kcal/mol) ± SD from the last 50-ns MD snapshots of acarbose in complex with human α-glucosidase estimated by MMPB/GBSA methods.

| Energy components | | Acarbose |
| --- | --- | --- |
| Gas phase | | |
| ΔE_electrostatic_ | -45.5081 ± 11.0314 | |
| ΔE_vdW_ | -33.2282 ± 3.9355 | |
| ΔE_gas_ | -78.7363 ± 11.1548 | |
| Solvation (GBSA) | | |
| ΔG_polar_ | 64.7375 ± 9.5427 | |
| ΔG_nonpolar_ | -5.7511 ± 0.4646 | |
| *ΔG_bind(MM/GBSA)_* | -19.7498 ± 4.5078 | |
| Solvation (PBSA) | | |
| ΔG_polar_ | 71.4422 ± 11.1595 | |
| ΔG_nonpolar_ | -6.6520 ± 0.3869 | |
| *ΔG_bind(MM/PBSA)_* | -13.9461 ± 5.9347 | |
